# 3D bioprinting of dynamic hydrogel bioinks enabled by small molecule modulators

**DOI:** 10.1126/sciadv.ade7880

**Published:** 2023-03-31

**Authors:** Sarah M. Hull, Junzhe Lou, Christopher D. Lindsay, Renato S. Navarro, Betty Cai, Lucia G. Brunel, Ashley D. Westerfield, Yan Xia, Sarah C. Heilshorn

**Affiliations:** ^1^ Department of Chemical Engineering, Stanford University, Stanford, CA, USA.; ^2^ Department of Materials Science and Engineering, Stanford University, Stanford, CA, USA.; ^3^ Department of Bioengineering, Stanford University, Stanford, CA, USA.; ^4^ Department of Chemistry, Stanford University, Stanford, CA, USA.

## Abstract

Three-dimensional bioprinting has emerged as a promising tool for spatially patterning cells to fabricate models of human tissue. Here, we present an engineered bioink material designed to have viscoelastic mechanical behavior, similar to that of living tissue. This viscoelastic bioink is cross-linked through dynamic covalent bonds, a reversible bond type that allows for cellular remodeling over time. Viscoelastic materials are challenging to use as inks, as one must tune the kinetics of the dynamic cross-links to allow for both extrudability and long-term stability. We overcome this challenge through the use of small molecule catalysts and competitors that temporarily modulate the cross-linking kinetics and degree of network formation. These inks were then used to print a model of breast cancer cell invasion, where the inclusion of dynamic cross-links was found to be required for the formation of invasive protrusions. Together, we demonstrate the power of engineered, dynamic bioinks to recapitulate the native cellular microenvironment for disease modeling.

## Teaser

Dynamic, viscoelastic bioinks are designed, optimized, and used to fabricate 3D models of breast cancer cell invasion.

## INTRODUCTION

Three-dimensional (3D) bioprinting is an emerging additive manufacturing technique with the potential to fabricate complex biological structures that mimic native tissue, opening the door for advances in personalized medicine, tissue regeneration, and pharmaceutical testing (*1*, *2*). As the most common bioprinting method, microextrusion printing uses pneumatic pressure or mechanical forces to dispense a bioink, here defined as a composite of both cells and polymer, from the print nozzle into a prespecified geometry (*3*, *4*). While advances in printing capabilities have enabled the construction of more geometrically complex architectures, the field remains limited by a lack of bioink materials with the mechanical properties suitable for both printing and subsequent cell culture (*5*, *6*). In general, a trade-off between printability and cell compatibility exists, in which stiffer, more viscous materials exhibit better shape fidelity after printing, while softer, less viscous materials provide suitable biophysical cues for maintaining cell viability and promoting cellular processes such as migration, proliferation, and differentiation (*7*, *8*). Therefore, a major challenge remains in designing bioinks that can both be printed into complex architectures and remain effective for 3D cell culture.

To create more biofunctional constructs, alternative bioinks are needed that better mimic the mechanical properties of native tissue. Most tissues within the body are viscoelastic and stress relaxing, such that they dissipate stress in a time-dependent manner following deformation (*9*). This is in contrast to many traditional materials used for 3D cell culture and bioprinting, which are typically cross-linked through irreversible covalent bonds and therefore exhibit elastic behavior. Recent work has demonstrated the importance of a material’s viscoelastic, time-dependent properties in regulating cell behavior and allowing for dynamic remodeling of the matrix through cell-generated forces (*10*–*14*). While advances within the tissue engineering community have led to the development of a number of dynamic, viscoelastic biomaterials with tunable properties for 3D cell culture, few of these systems have been translated into bioinks (*15*).

Here, we design a dynamic bioink system to address the need for engineered, tunable matrices that exhibit viscoelastic behavior to fabricate 3D bioprinted tissue models. We use dynamic covalent bonds, which can spontaneously break and reform under physiological conditions, as the cross-linking mechanism (*16*). Hydrogels cross-linked by dynamic covalent bonds have been shown to be viscoelastic, shear thinning, and self-healing (*17*–*22*). Unfortunately, dynamic covalent cross-links also make the materials prone to erosion and viscous flow over time, making them very challenging to use for 3D printing. To overcome this challenge, light-induced curing has previously been used, with the undesirable effect of altering the viscoelastic mechanics of the hydrogel by introducing static covalent cross-links into the network (*23*, *24*). As an alternative approach, we develop a strategy that uses two small molecules, a catalyst and a competitor, to dynamically modulate the cross-linking kinetics and degree of network formation, respectively. These diffusive small molecules allow separate fine-tuning of the network dynamics pre-printing and post-printing, resulting in a bioink that initially has viscoelastic properties appropriate for printing and then acquires the ideal viscoelastic properties for long-term cell culture. Critically, this independent control of the bioink mechanical properties before and after printing enables bioinks to be optimized for both printability and cell culture. Because it is well established that a cell’s phenotype is dependent on matrix properties, independent control of the post-printing properties will aid in tailoring a bioink’s final matrix mechanics to the cell type of interest to improve the biofunctionality of printed constructs.

3D bioprinting holds promise for the rapid fabrication of physiologically relevant in vitro disease models for drug screening. In particular, breast cancer progression is known to be governed by cell-matrix interactions and involve substantial matrix remodeling in vivo, which cannot be recapitulated in traditional 2D culture models (*25*, *26*). While 3D in vitro models such as organoids and organ-on-a-chip devices can more closely mimic the tumor microenvironment (*27*, *28*), they lack the ability to precisely control the spatial organization of different cellular and material components. Therefore, we leveraged our dynamic bioink system to print 3D models of breast cancer invasion and investigated how spatial patterning of different biochemical features affected cell phenotype. We found that both dynamic covalent bonds and integrin engagement were required to allow cell invasion through the bioink. Together, these results illustrate the need for dynamic bioink materials that better recapitulate native tissue mechanics and demonstrate the power of kinetic control over the cross-linking reaction to enable both printability and stability of dynamic materials.

## RESULTS

### Design of a dynamic bioink system with tunable material properties

**Fig. 1.**
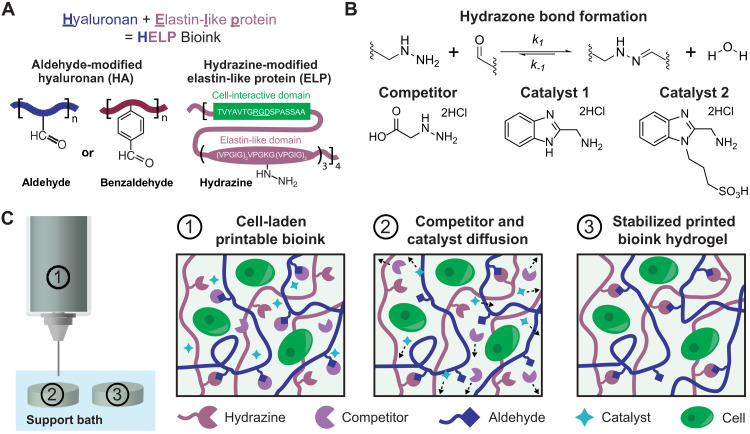
HELP bioinks are engineered, dynamic hydrogels whose printability can be tuned through the addition of small molecules. (**A**) Schematic of the engineered HELP bioink, which consists of HA modified with either an ALD or BZA group and an ELP modified with HYD groups. (**B**) When the HA and ELP components are mixed, they form hydrazone bonds. The printability of these hydrazone-based inks is modulated by introducing two small molecules: a competitor and a catalyst. The competitor binds to free aldehydes present in the bioink, reducing the number of cross-links present in the print syringe. The catalyst increases the rate of bond exchange. Catalyst 1 was used for all acellular experiments and catalyst 2 for all cellular experiments. (**C**) The two polymer components, the competitor, and the catalyst are mixed with cells to form a bioink. The bioink is loaded into the print cartridge and printed into a gel support bath. Following printing, the small molecule competitor and catalyst can diffuse away from the printed structure into the support bath while the ink remains in place, stiffening and stabilizing the bioink.

To produce viscoelastic, dynamic bioinks, we chose to use hydrazone cross-linking, a reversible bond type between aldehyde (ALD) and hydrazine (HYD) functional groups. Hydrazone chemistry is well suited as a cross-linking mechanism to form hydrogels for cell encapsulation as it is cytocompatible, is reversible under physiological conditions, and has only water as a by-product (*29*). The dynamic nature of the hydrazone bond also allows the material to be viscoelastic and stress relaxing (*17*–*21*). To prepare these inks, we modified hyaluronan (HA) with ALD or benzaldehyde (BZA) functional groups and an elastin-like protein (ELP) with HYD functional groups to create a hyaluronan elastin-like protein (HELP) matrix (Fig. 1A and figs. S1 and S2). HA is a linear polysaccharide found in many tissue types and is commonly used in tissue engineering due to its cell compatibility, relative ease of chemical modification, and important role in many biological processes, including cancer progression (*30*). ELP is a recombinant, engineered protein containing repeating elastin-like and bioactive domains (*31*). The elastin-like region consists of amino acid sequences derived from elastin, and the bioactive domain can be designed to contain different cell-binding domains, including an Arg-Gly-Asp (RGD) integrin-binding motif (*32*, *33*). When mixed, these two polymer components form hydrazone bonds, which can break and reform under physiological conditions (Fig. 1B). This results in a dynamic, tunable, fully chemically defined, and reproducible material system. Previously, these materials have been reported to support the culture of various cell types, including chondrocytes, mesenchymal stromal cells, and human intestinal organoids (*19*, *34*, *35*). Despite its viscoelastic properties and suitability for cell culture, the HELP material has not previously been printed. Like many dynamically cross-linked materials, it remains difficult to extrude as a continuous filament, likely due to the slow dissociation rate of the hydrazone bond and the relatively large molecular weights of HA and ELP (100 and 37 kDa, respectively) that can lead to polymer entanglements (*23*, *36*, *37*).

To increase the printability of HELP inks, we introduce two small molecules: a competitor and a catalyst. Here, we chose a glycine-based hydrazine analog as a small molecule competitor that can disrupt hydrazone bond formation (Fig. 1B). This molecule was chosen because it was not cytotoxic to cells (fig. S3), unlike some other hydrazine-containing molecules (*38*). The competitor can reversibly react with aldehyde groups present on the functionalized HA, which reduces the number of cross-links in the hydrogel and thus reduces the overall stiffness of the ink. The catalyst increases the rate of hydrazone bond exchange, which has been shown to increase shear thinning in other hydrogel systems (*39*). Catalyst 1 (a commercially available benzimidazole-based catalyst previously demonstrated to efficiently accelerate hydrazone formation; Fig. 1B) was used for all acellular studies. Catalyst 2 (a sulfonated derivative of catalyst 1; Fig. 1B) was used for all cellular studies because its zwitterionic structure was found to be more cytocompatible while remaining as efficient as the original catalyst 1 (*39*). Together, the competitor and catalyst enable the ink to be readily extruded during printing. The ink is printed into a support bath, which physically confines the ink as it is being deposited and eliminates the requirement that the ink be self-supporting in air. This freeform printing technique has previously been shown to enable printing of very soft hydrogel materials (*40*–*42*). Here, the support bath is composed of gelatin microparticles, but this method for printing dynamic covalent inks should be compatible with other support baths. Following extrusion, a cross-linking step is typically used to stabilize the printed structure before the bath is removed, a process also referred to as “curing.” To date, two primary methods have been used to initiate curing: use of external triggers such as light or heat (*43*); or the diffusion of small-molecule cross-linkers, catalysts, or ions into the ink material (*44*–*47*). Here, we introduce an alternative strategy: the diffusion of the catalyst and competitor out of the ink material to increase ink cross-linking, resulting in a stiffened and stabilized printed construct (Fig. 1C).

**Fig. 2.**
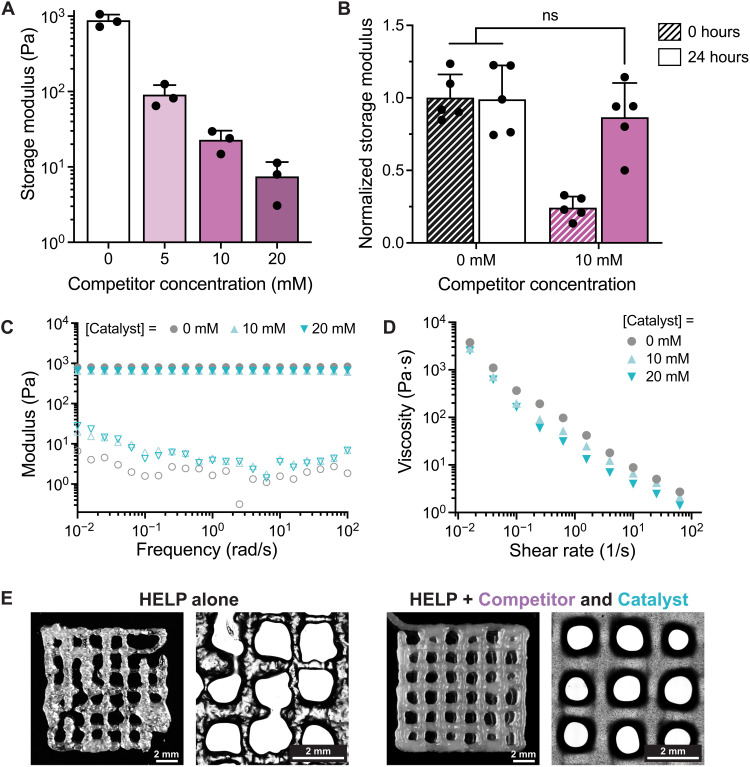
The addition of a small molecule competitor and catalyst improves HELP ink printability. (**A**) Addition of the competitor decreases the storage modulus of the HELP ink in a dose-dependent manner (*n* = 3, means ± SD). (**B**) The shear moduli of HELP hydrogels with and without competitor are measured after 0 and 24 hours in PBS (*n* = 5, means ± SD). Following 24 hours in PBS, the storage moduli of HELP hydrogels containing competitor recover to those of non–competitor-containing gels, such that no statistical difference was measured [ordinary one-way analysis of variance (ANOVA) with Tukey multiple comparisons correction. ns, not significant. (**C**) Frequency sweeps of HELP hydrogels with varying amounts of catalyst show that changing catalyst concentration does not affect hydrogel stiffness. (**D**) The ability of HELP hydrogels to shear thin increases with increasing amounts of catalyst. (**E**) Representative bright-field images of printed lattices of HELP alone and the HELP ink containing competitor and catalyst.

First, we explored the effects of the small molecule competitor on HELP hydrogel mechanical properties. Without the competitor and catalyst, mixing HA-ALD and ELP-HYD at 1 wt % produced hydrogels with storage moduli on the order of 1000 Pa. Addition of the small molecule competitor decreased the storage modulus in a dose-dependent manner, up to two orders of magnitude (Fig. 2A). While the addition of competitor can also increase the time required for gelation, hydrogels containing the highest amount of competitor (20 mM) still formed gels within 30 min (fig. S4). This sets a lower bound for the time needed to allow for gelation in the print cartridge before printing. While the competitor decreases the stiffness of the ink in the print cartridge, the reversibility of its binding to the network should allow it to passively diffuse away from the printed structure and into the aqueous medium following printing, thus stiffening the ink. To test this, we cast HELP hydrogels with and without 10 mM competitor. We measured their rheological properties before the addition of any aqueous medium and again after 24 hours in phosphate-buffered saline (PBS), when the network should be at equilibrium (Fig. 2B). The initial modulus of the hydrogels containing competitor is reduced by approximately fourfold compared to that of hydrogels without competitor. However, after 24 hours in PBS, the storage moduli of the HELP hydrogels containing competitor recovered to those of non–competitor-containing gels. This indicates that the competitor can unbind and diffuse away from the hydrogel, stiffening the HELP ink into a more robust hydrogel.

Next, we investigated how the catalyst affects the gel’s rheological properties. Because the catalyst only accelerates the rate of bond exchange but does not affect the reaction equilibrium, the gel network structure should remain unchanged. When we perform frequency sweeps of HELP hydrogels with and without catalyst, we see that the plateau storage modulus, which is related to the ink stiffness, is not affected by the addition of the catalyst (Fig. 2C). In contrast, increasing the catalyst concentration increases the gels’ ability to shear thin, as indicated by a steeper drop in viscosity with increasing shear rate (Fig. 2D). Therefore, incorporation of the catalyst into our bioink design should allow the material to be extruded more easily upon the application of a mechanical force.

To improve the printability of our dynamic ink, we combined both the competitor and catalyst with our engineered HELP material. To form an ink, we first mix the competitor and catalyst with ELP-HYD so that all hydrazine functional groups are present in the same solution. We then add in HA-ALD and load the material into a print syringe. Before printing, we allow a hydrogel network to form within the print syringe over 30 min. Using this procedure, we first print HELP alone (1 wt % HA-ALD/1 wt % ELP-HYD) into a 10 mm–by–10 mm lattice structure. While the material can be extruded, the printed filaments are fractured and deposited inconsistently, which is not ideal for 3D bioprinting of complex structures (Fig. 2E, left). Incorporation of both the competitor (20 mM) and catalyst (10 mM) decreases the ink stiffness and increases shear thinning without changing the ink’s stress relaxation rate (fig. S5). Therefore, with the addition of the competitor and catalyst, the ink no longer fractures during extrusion, and printed filaments are smooth and consistently deposited (Fig. 2E, right).

While we used the HELP material as a representative ink to demonstrate the advantages of using a small molecule competitor and catalyst to modulate printability, this strategy can also be extended to other polymer systems. We additionally functionalized gelatin, an example of a naturally derived polymer, and polyethylene glycol (PEG), an example of a synthetic polymer, with hydrazine and aldehyde moieties. Mixtures of PEG-HYD/PEG-ALD, gelatin-HYD/PEG-ALD, and gelatin-HYD/HA-ALD could also form stiff hydrogels (>1000 Pa) without competitor, and addition of competitor reduced their stiffness in a dose-dependent manner (fig. S6). Guided by these rheological measurements, we were then able to select suitable amounts of competitor and catalyst for each hydrogel system to form a library of hydrazone-based inks. Each of these hydrazone inks (PEG-HYD/PEG-ALD, gelatin-HYD/PEG-ALD, and gelatin-HYD/HA-ALD) was printable with the addition of competitor and catalyst and could be used to fabricate complex structures when printed into a gel support bath (fig. S6D).

### Optimization of HELP ink materials to improve printability and long-term stability

**Fig. 3.**
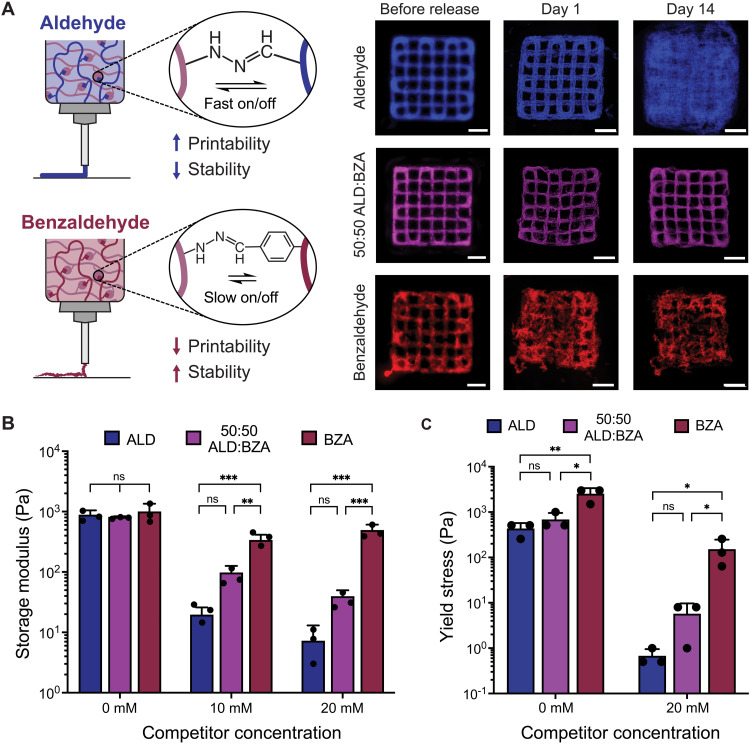
HELP ink materials can be optimized to improve printability and long-term stability. (**A**) Schematic depicting that the HA component of HELP can be modified with either an ALD group (faster bond exchange rate) or a BZA group (slower bond exchange rate). Representative images of printed lattices show that changing the ratio of ALD:BZA affects ink printability and the stability of printed structures over time. Scale bars, 2 mm. (**B**) Tuning the ratio of ALD:BZA does not affect the storage modulus of HELP hydrogels. The addition of competitor has a smaller effect on the storage modulus of BZA-only HELP hydrogels as compared to that of ALD-only hydrogels and ALD:BZA blends (*n* = 3, means ± SD, ordinary one-way ANOVA with Tukey multiple comparisons correction, **P < 0.01, ***P < 0.001). (**C**) Yield stress behavior changes as a function of cross-linking kinetics (i.e., ALD-only, 50:50 ALD:BZA, and BZA-only) and with the addition of the competitor (*n* = 3, means ± SD, ordinary one-way ANOVA with Tukey multiple comparisons correction, *P < 0.05, **P < 0.01). ns, not significant.

Based on our dynamic bioink platform, we further optimized the HELP ink formulation to improve the stability of printed constructs over time, which is a key requirement for long-term cell culture (e.g., to allow for multiday proliferation, differentiation, or migration of cells). One challenge associated with using dynamic cross-links that has precluded their inclusion in most bioink designs is their propensity for erosion, swelling, and creep behavior (*15*). To optimize the stability of HELP inks, we first printed a HELP formulation containing 1 wt % HA-ALD, 1 wt % ELP-HYD, 10 mM catalyst, and 20 mM competitor into a standard lattice structure within a commercially available support bath composed of gelatin microparticles. We show that the material is highly printable and that the printed lattice can be successfully released from the support bath (Fig. 3A). Following printing, we allow the structures to remain in the support bath for 1.5 hours at room temperature (RT) to allow the competitor and catalyst to diffuse away, thus stabilizing the structure. Because the gelatin support bath is thermoreversible, it can be melted away at 37°C, allowing the stabilized structure to be recovered, washed with PBS, and cultured in any aqueous medium. While the lattice’s integrity is maintained immediately after release from the support bath, the structure then experiences substantial swelling and erosion within 1 day of being kept at 37°C in PBS, and the printed structure is no longer discernible after 14 days (Fig. 3A, top row). Further measurement of gel erosion kinetics showed that 27% of the printed structure had eroded after 1 day, and 47% had eroded after 14 days (fig. S7). Therefore, this HELP ink formulation (HA-ALD/ELP-HYD) is suitable for printing complex, temporary structures but not for printing structures that need to be cultured long term.

To improve the stability of hydrazone–cross-linked materials, secondary polymer networks with static covalent bonds, such as light-curable networks, have previously been used to decrease hydrogel erosion, with the adverse effect of altering the material’s viscoelastic properties (*23*). To retain the ability of the HELP material to stress relax and to avoid the use of static bonds, we sought to modulate stability in an alternative manner. We hypothesized that the relatively fast exchange dynamics between HA-ALD and ELP-HYD may contribute to gel erosion. Therefore, we synthesized an alternative benzaldehyde-modified HA (HA-BZA) because the BZA group has a slower bond exchange rate than the ALD. We altered the degree of functionalization of HA-BZA such that the storage modulus of HELP hydrogels formulated with HA-BZA matched that of hydrogels formulated with HA-ALD (fig. S2). The ink containing HA-BZA (1 wt % HA-BZA/1 wt % ELP-HYD) resulted in printed lattices that maintained their shape fidelity over 2 weeks in culture and eroded less than 3% (fig. S7), validating our hypothesis that slower bond exchange kinetics would lead to less erosion. Unfortunately, the HA-BZA ink had poor printability, even with much higher amounts of competitor and catalyst (up to 80 mM competitor and 50 mM catalyst; Fig. 3A, bottom row).

To strike a balance between the BZA’s poor printability but good long-term stability and the ALD’s good printability but poor stability, we blended the two materials together in a 50:50 ALD:BZA ratio to create an ink composed of 1 wt % ELP-HYD, 0.5 wt % HA-ALD, and 0.5 wt % HA-BZA. Blending these two HA materials together and adding similar amounts of competitor (20 mM) and catalyst (10 mM) as for the ALD-only HELP ink led to improved structural stability over ALD-only inks while maintaining good printability (Fig. 3A, middle row). The erosion rate was found to be similar to that of BZA-only and considerably less than that of ALD-only inks (fig. S7). We used this optimized 50:50 ratio of ALD:BZA for all remaining studies.

Next, we determined whether the observed printability of these materials was related to the gels’ rheological properties. We measured the storage moduli of ALD-only, BZA-only, and 50:50 ALD:BZA-containing HELP gels at the same polymer weight percentage (1 wt %) and found that the stiffness was not significantly different among the three groups, indicating that the degree of network formation was similar (Fig. 3B). However, the reduction in storage modulus with increasing competitor concentration was much smaller in BZA-only inks as compared to that in ALD-only and the optimized 50:50 ALD:BZA inks. This is presumably due to the slower dissociation kinetics of the BZA compared to that of the ALD, which may reduce efficient exchange between the competitor and the hydrogel network and prevent an equilibrium network from forming at shorter time scales (<1 hour). Next, we measured the yield stress of HELP materials with and without 20 mM competitor. Yield stress, defined as the stress required to initiate flow, is related to the amount of force required to extrude material through the syringe during printing. When the yield stress is too high, the ink material may not be printable or could fracture during extrusion. Without the competitor, we find that the yield stress of all three HELP blends (ALD-only, 50:50 ALD:BZA, and BZA-only) is relatively high (around 1000 Pa) and that the yield stress of the BZA-only ink is significantly higher than that of the other two materials (Fig. 3C). Upon addition of the competitor, the yield stress is reduced for all three conditions but remains much higher in the BZA-only ink as compared to that in the ALD-only and 50:50 ALD:BZA inks. ALD-only and the optimized 50:50 ALD:BZA blend had similar yield stresses, corresponding well with our observation that both materials were printable into consistent and intact lattice structures within a gel-phase support bath. Together, these results indicate that both stiffness and yield stress can be used as predictors for bioink printability, where higher stiffness and higher yield stress lead to decreased printability for a given bioink formulation.

### Use of dynamic materials to model the cancer microenvironment

Our primary goal in developing these dynamic bioinks was to use them to model dynamic processes that occur in native tissue. One process known to involve extensive cellular remodeling of the extracellular matrix (ECM) is cancer invasion; therefore, we sought to use the optimized dynamic HELP ink to create models of the cancer microenvironment. In particular, ECM mechanics are an important mediator of breast cancer cell invasion, where matrix composition, architecture, and stiffness have been shown to correlate with tumor aggressiveness and metastasis (*48*–*51*). Furthermore, recent studies have also suggested that cancer cells may respond to the stress relaxation rate of viscoelastic matrices (*52*, *53*). While matrix viscoelasticity is known to affect cell spreading (*54*), differentiation (*10*), and migration (*55*), less is known about how viscoelasticity may affect cancer cell invasive behavior (*56*). Therefore, we first tested how premalignant breast cancer cells (MCF10AT) responded to being cultured within HELP.

**Fig. 4.**
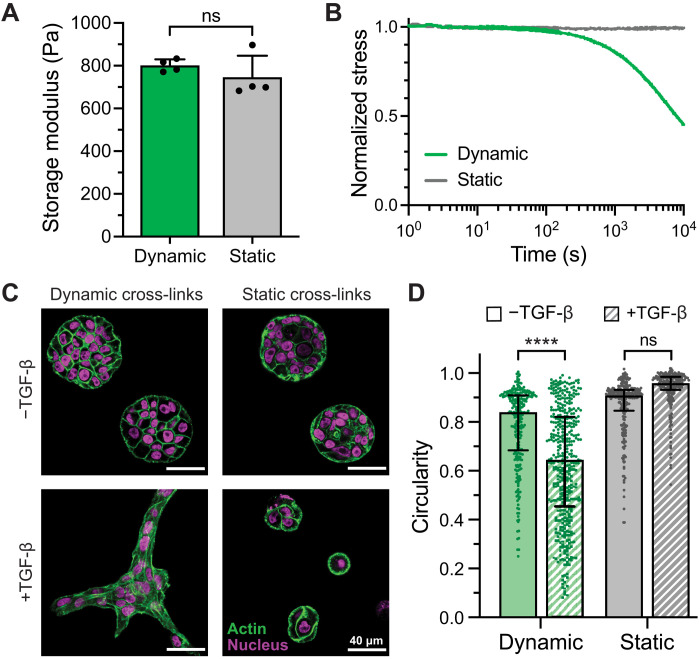
Dynamic covalent cross-links enable breast cancer cell invasion upon addition of TGF-β, while static cross-links do not. (**A**) The storage moduli of HELP hydrogels cross-linked by dynamic covalent and static covalent chemistries can be matched. The polymer composition and weight percentage are the same for both HELP materials (1% HA and 1% ELP). (*n* = 4, means ± SD, unpaired two-tailed Student’s *t* test). (**B**) Stress relaxation of HELP hydrogels with dynamic and static covalent cross-links. (**C**) Representative immunofluorescence images of MCF10AT cells grown in HELP-dynamic and HELP-static for 6 days with and without the addition of TGF-β, a known mediator of breast cancer cell invasion. (**D**) MCF10AT cell cluster circularity in dynamically and statically cross-linked HELP materials after 6 days (data from one representative biological replicate experiment with median and interquartile range shown, *n* = 239 to 423 cell clusters, two-tailed Mann-Whitney test, ****P < 0.0001, *N* = 3 biological replicates). ns, not significant.

To determine whether matrix viscoelasticity plays a role in breast cancer cell invasion, we synthesized an alternative version of HELP that was cross-linked by static covalent bonds (HELP-static) rather than dynamic covalent bonds (HELP-dynamic). To do so, we modified HA with tetrazine functional groups and ELP with norbornene functional groups (figs. S8 and S9). Upon mixing, these two polymer components also form hydrogels through the tetrazine-norbornene “click” reaction. However, this reaction is irreversible under aqueous conditions, unlike the dynamic, reversible hydrazone bond. By controlling the degree of polymer functionalization, we matched the HELP-static and HELP-dynamic materials such that they had the same stiffness at a given polymer weight percentage (Fig. 4A). Thus, these two materials have identical HA content, ELP content, and stiffness but vary in their cross-linking chemistry. This difference leads to changes in viscoelastic behavior, where the HELP-dynamic materials are stress relaxing, while the HELP-static materials do not dissipate stress over time (Fig. 4B).

We then encapsulated MCF10AT cells as single cells within these two HELP materials. After 6 days in culture, the cells formed noninvasive spheroids in both materials, consistent with previous reports of MCF10AT spheroid formation in 3D Matrigel and collagen cultures (Fig. 4C, top) (*57*, *58*). While the HELP-static materials are not stress relaxing, cells can still secrete enzymes that locally degrade the HELP matrix, allowing for spheroid formation (*34*, *51*). To produce models of cell invasion, we then treated the cells with transforming growth factor–β (TGF-β), a known regulator of epithelial-to-mesenchymal transition (EMT) in breast tumors, which is thought to promote a more invasive phenotype (*51*, *59*). Directly after encapsulation, we supplemented the medium with TGF-β (10 ng/ml) for cultures in HELP-dynamic and HELP-static materials and allowed morphogenesis to proceed for a period of 6 days. Using immunofluorescence confocal microscopy, we observed notable differences in cell cluster morphology with the addition of TGF-β depending on the type of cross-linking chemistry (Fig. 4C, bottom). TGF-β drove invasion only in HELP-dynamic, resulting in cell clusters with large protrusions into the surrounding matrix. In HELP-static, treatment with TGF-β produced smaller, mostly noninvasive cell clusters (fig. S10). Quantification of cell cluster circularity confirmed that in the absence of TGF-β, both materials supported proliferation of MCF10AT cells to form highly circular, noninvasive spheroids (Fig. 4D). Upon addition of TGF-β, cells encapsulated within HELP-dynamic formed clusters that were significantly less circular (median circularity of 0.64, where 1 is a perfect circle) than those without TGF-β (median circularity of 0.84). In contrast, circularity was not significantly different for cells grown in HELP-static with or without TGF-β (median circularities of 0.92 and 0.96, respectively). Consistent with the morphological observation that cells undergo greater invasion in the dynamic gels upon addition of TGF-β, gene expression analysis revealed that cells cultured in HELP-dynamic gels supplemented with TGF-β had significantly lower levels of E-cadherin expression compared to those cultured in HELP-static, indicative of the loss of cell-cell adhesion, one of the signatures of EMT (*P* < 0.001, fig. S11). Similarly, expression of vimentin, a marker for a more mesenchymal phenotype, was significantly higher (*P* < 0.05) in HELP-dynamic compared to that in HELP-static. Other common EMT markers [N-cadherin, SNAIL1, and Zinc finger E-box binding homeobox 1 (ZEB1)] were up-regulated in both HELP-dynamic and HELP-static upon addition of TGF-β, but expression levels were not significantly different between the two matrices (fig. S11). Our morphological findings and gene expression analysis suggest that matrix viscoelasticity is required in our model system to permit growth factor–induced cell invasion. This is consistent with previous reports that increasing covalent cross-linking density restricts invasive protrusions in breast cancer cells through mechanical confinement (*60*).

### 3D bioprinting of dynamic bioinks to form spatially patterned constructs

**Fig. 5.**
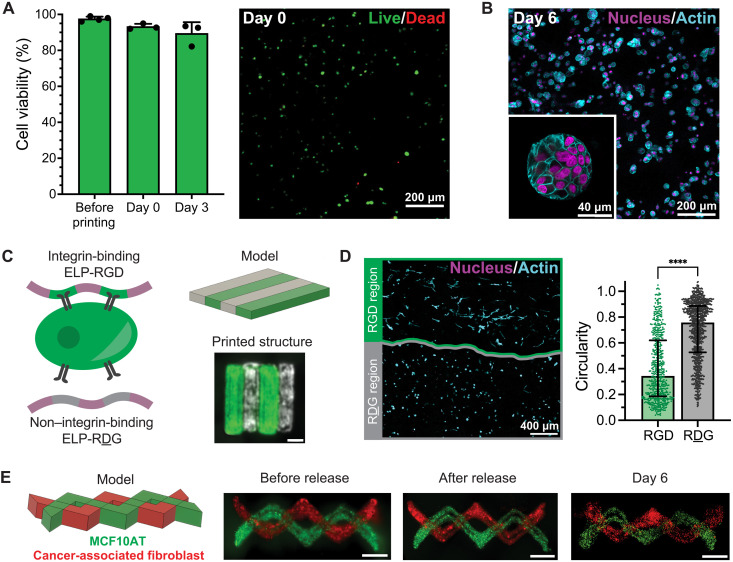
HELP bioinks can be used to print models of the breast cancer microenvironment. (**A**) MCF10AT cells in printed HELP bioinks retain high viability immediately after printing and after 3 days in culture as tested by a Live/Dead cytotoxicity assay (*n* = 3 to 4, means ± SD). (**B**) Representative images of MCF10AT cells in printed HELP bioinks, which form noninvasive spheroids after 6 days in culture. (**C**) Schematic of the recombinant ELP component of HELP, which can be engineered to contain a non–cell-interactive scrambled RDG sequence instead of the integrin-binding RGD sequence. Alternating regions of HELP-RGD (green, fluorescent microspheres) and HELP-RDG (gray, fluorescent microspheres) can be printed together to form a cohesive structure. Scale bar, 2 mm. (**D**) Printed MCF10AT cells treated with TGF-β are significantly less circular in printed HELP-RGD regions compared to that in HELP-RDG regions (*N* = 3 replicate printed structures, *n* = 92 to 448 cells per printed region, median ± interquartile range, two-tailed Mann-Whitney test, ****P < 0.0001). (**E**) Printed structures containing both MCF10AT cells (green, dyed with CellTracker Green) and cancer-associated fibroblasts (CAFs; red, dyed with CellTracker Red) maintain their spatial patterning in the support bath (before release), after release from the support bath (after release), and after 6 days in coculture. Scale bars, 2 mm.

We next bioprinted the optimized HELP material with cells to fabricate multimaterial constructs to evaluate how matrix properties affect breast cancer cell phenotype. We formed the bioink by resuspending pelleted cells in ELP-HYD, then adding competitor and catalyst, and lastly mixing in a 50:50 blend of HA-ALD/HA-BZA. The presence of cells was not found to affect the gelation kinetics or final modulus of HELP inks (fig. S12). Therefore, before printing, we allowed a hydrogel network to form within the print syringe for 30 min and used the same print parameters as for acellular prints. Following printing into a support bath, we allowed the competitor and catalyst to diffuse away from the printed structure for 1.5 hours before removing the printed construct from the bath, washing it with PBS, and placing it in cell culture medium. We found that HELP bioinks containing cells were highly printable, consistent with our results when printing acellular constructs (fig. S13). We first confirmed that MCF10AT cells remained viable throughout the printing process. Using a Live/Dead cytotoxicity assay, we found that cell viability was similar before and after this printing process and remained high after 3 days in culture (Fig. 5A). After 6 days in culture, MCF10AT cells were able to proliferate and form noninvasive spheroids, similar to their behavior in cast hydrogel cultures, and the spheroids were well distributed throughout the printed construct (Fig. 5B).

One key advantage of fabricating structures using 3D bioprinting is that it allows for spatial patterning of different biochemical features and cell types within a single matrix. In the tumor microenvironment, dysregulation of integrin signaling, altered deposition and degradation of matrix components, and localized changes in matrix biochemistry can create distinct spatial compartments within the tumor (*61*). 3D bioprinting can be used to recapitulate some of these biochemical and biophysical changes to the matrix in a spatially defined manner (*62*). To demonstrate how different matrix biochemistries can be integrated into separate regions of bioprinted constructs, we printed alternating regions of HELP with and without cell-adhesive ligands (Fig. 5C). As a protein-engineered material, ELP can be designed to include a fibronectin-derived RGD domain, which is a ligand for integrin receptors on the cell surface. Alternatively, ELP can be designed to include a non–integrin-binding, scrambled Arg-Asp-Gly (RDG) sequence as part of its polymer backbone without altering the rest of the protein. This ELP variant can be chemically modified to display hydrazine groups that dynamically cross-link with aldehyde-modified HA to form an RDG ink. This RDG ink has identical mechanical properties and HA composition as the RGD-containing HELP material (*34*). MCF10AT cells and fluorescent microspheres were suspended within each of the two bioink materials (either containing RGD or RDG peptides), and then the two bioink materials were printed side by side using separate nozzles. The two materials formed a single cohesive structure with distinctive boundaries, as determined by imaging the two different fluorescent microspheres contained within each of the two inks. The printed construct was then treated with TGF-β for 6 days in culture. After 6 days, cells printed within RGD regions formed invasive structures with significantly lower circularity (median of 0.34) than those printed within RDG regions (median of 0.76, *P* < 0.0001) (Fig. 5D). This difference in cell circularity was also observed when cells were separately cast in RGD- and RDG-containing HELP hydrogels without bioprinting (fig. S14). These data indicate that integrin binding assists with TGF-β–induced cell invasion and that paracrine signaling alone is not sufficient to stimulate cell invasion. These observations are consistent with current models of breast cancer invasion that suggest that integrin-based interactions with the matrix microenvironment play a dominant role in cancer progression (*63*, *64*). Together, this example demonstrates how 3D bioprinting can be leveraged to promote differential cell responses within the same printed, viscoelastic structure.

HELP bioinks can also be used to pattern multiple cell types together in the same structure to create more complex models of the tumor microenvironment. Cancer-associated fibroblasts (CAFs) and MCF10AT cells were each used to form a bioink from the optimized HELP ink material, and then the two bioinks containing either cell type were printed into a single structure using two separate nozzles. The two materials formed a cohesive structure with overlapping regions containing the two cell types in contact with each other, as well as regions of spatial confinement of each individual cell type (Fig. 5E). The structural integrity of this printed geometric pattern was maintained over 6 days in culture. This highlights that a single formulation of HELP can be used to support the culture of multiple different cell types, which, in future studies, could be leveraged to study the dynamic interactions between tumor and stromal cells over time.

## DISCUSSION

The field of 3D bioprinting is limited by a lack of suitable bioink materials that recapitulate native tissue mechanics. In particular, 3D bioprinting has the potential to enable rapid fabrication of models of the tumor microenvironment for high-throughput testing of new therapeutics, but viscoelastic materials that allow for cellular remodeling of the ECM are needed to mimic the highly dynamic processes involved in tumor progression. However, dynamic materials can be challenging to print due to the common trade-off between printability and long-term stability. Here, we introduced a strategy to create printable, dynamic bioinks by using temporarily present small molecule competitors and catalysts to control bioink mechanical properties before and after printing. We used the competitor to decrease the number of cross-links in the material within the print syringe and the catalyst to alter the bond exchange kinetics. This formulation resulted in a soft, shear-thinning material that was easy to extrude and formed consistent filaments. The printed structure could then be stabilized in place through the diffusion of the catalyst and competitor out of the hydrogel network.

To improve the long-term stability of printed constructs, we further changed the molecular structure of the matrix-bound reaction constituents to modulate the rate of the hydrazone bond exchange reaction. Inclusion of a BZA moiety, which has a slower bond exchange rate than an ALD functional group, allowed us to increase the stability of printed structures for up to 2 weeks while preserving printability. This strategy avoids use of a secondary network of static covalent bonds, which has previously been shown to increase stability at the expense of altering the material’s viscoelastic, stress-relaxing behavior (*23*, *24*). While this demonstration used the HELP material (composed of HA and ELP and cross-linked through hydrazone chemistry) as a representative bioink, the strategies introduced here can readily be extended to other polymer systems, including both naturally derived polymers such as gelatin and synthetic polymers such as PEG. Thus, our results demonstrate a generalizable approach for printing dynamic, viscoelastic materials by leveraging small molecule competitors and catalysts.

To demonstrate its applicability for mimicking biological processes, we then used our engineered, dynamic material to create models of breast cancer invasion. We found that both matrix viscoelasticity and integrin engagement were required for growth factor–induced cell invasion. Breast cancer cells cultured within HELP materials with dynamic covalent bonds formed invasive structures with multiple protrusions upon the addition of TGF-β, while cells cultured in control gels with static covalent bonds remained highly spherical. This suggests that viscoelastic materials are needed to recapitulate dynamic processes such as cancer invasion in 3D bioprinted models. Next, we used the HELP bioink platform to pattern spatially distinct regions of the construct with different biochemical features. Stark differences in cell spreading were observed in regions containing the integrin-binding (RGD) peptide compared to the non–integrin-binding (RDG) regions. This indicates that integrin engagement is also necessary for growth factor–induced invasion and demonstrates how the HELP bioink platform can be used to elicit differential cell responses in spatially distinct regions within the same printed construct. In addition, multiple cell types could be printed together to form a single cohesive structure that maintained its structural integrity over time. This sets the stage for future studies to investigate how spatial distribution of other biochemical features, cell types, and material properties affect disease progression within biomimetic, viscoelastic matrices.

In summary, we have developed a dynamic, viscoelastic bioink material that can allow for dynamic changes within bioprinted models. This approach uses small molecules to fine-tune the reaction kinetics and degree of hydrogel network formation to enable control of material properties in the print syringe and thus improve ink printability. Separately, the final material properties of the structure can be dynamically modified after printing, where diffusion of the small molecule catalyst and competitor out of the printed structure stiffens and stabilizes the construct. This generalizable strategy could be readily applied to other dynamic bioinks to improve printability and long-term stability. Applying this strategy with the HELP bioink system, we demonstrated that both viscoelastic mechanical behavior and the inclusion of integrin-binding peptides were required to allow breast cancer cell spreading in response to TGF-β in 3D bioprinted models. We envision that the dynamic bioinks and control strategies developed here will provide new opportunities for printing tissue-engineered constructs and complex in vitro models.

## MATERIALS AND METHODS

### Synthesis of hydrazine-functionalized ELP

The recombinant ELP was expressed in *Escherichia coli* and purified as previously reported (*31*). ELP (37 kDa) includes 14 primary amines and either a cell-adhesive RGD peptide sequence or a scrambled, non–cell-interactive RDG sequence (*65*). ELP-HYD was synthesized as described previously (*17*, *19*, *34*). The following protocol is written for a 1-g batch but can be scaled as needed. Briefly, ELP was dissolved in 13.66 ml of anhydrous dimethyl sulfoxide (DMSO; Sigma-Aldrich) to a concentration of 7.3 wt %. Then, an equal volume of anhydrous dimethylformamide (DMF; Sigma-Aldrich) was then added to the same vessel. Separately, hexafluorophosphate azabenzotriazole tetramethyl uranium (HATU; 281 mg, 0.738 mmol, 2 equiv per ELP amine; Sigma-Aldrich) and tri-Boc hydrazinoacetic acid (303 mg, 0.775 mmol, 2.1 equiv per ELP amine; Sigma-Aldrich) were dissolved in 13.66 ml of DMF. To this mixture, 4-methylmorpholine (203 μl, 0.92 g/ml, 1.8 mmol, 5 equiv per ELP amine; Sigma-Aldrich) was added, and the reaction was allowed to proceed for 10 min at RT. This activated tri-Boc hydrazinoacetic acid mixture was added to the ELP reaction vessel, and the reaction was allowed to proceed overnight at RT. The next day, the Boc-protected intermediate was precipitated using ice-cold diethyl ether (~120 ml; Thermo Fisher Scientific), collected via centrifugation (12,000 rpm for 10 min at 4°C), and then dried overnight. The Boc groups were then removed (hydrazine deprotected) by resuspending the dried pellet in 6 ml of a 50:50 mixture of dichloromethane (Sigma-Aldrich) and trifluoroacetic acid (Sigma-Aldrich) and stirring for 4 hours at RT. The product (ELP-HYD) was then precipitated in chilled diethyl ether and collected via centrifugation (12,000 rpm for 10 min at 4°C). The pelleted final product was allowed to dry completely and then resuspended in water. The solution was then dialyzed against Milli-Q water for 3 days at 4°C with changes at least twice per day (molecular weight cutoff, 10 kDa), sterile-filtered through a 0.22-μm filter, and lastly lyophilized to produce a white solid, which was stored at −20°C. A fluorescently labeled version (ELP-HYD-Cy5), used in gel erosion (fig. S7) and printability studies (fig. S13), was synthesized via the reaction of an *N*-hydroxysuccinimide (NHS) ester (Cy5 NHS ester, Lumiprobe) with the remaining primary amines present on ELP-HYD.

### Synthesis of norbornene-functionalized ELP

First, the hydrophilicity of ELP was altered through conjugation of a PEG oligomer via an amidation reaction. Briefly, lyophilized ELP was dissolved in anhydrous DMSO at 7.5 wt % and allowed to stir for 10 min before adding an equal volume of anhydrous DMF and stirring for an additional 10 min. In a separate flask, acid-PEG_12_-NHBoc (2 equiv per ELP amine; BroadPharm) was dissolved in the same volume of DMF used to dissolve the ELP. Once dissolved, HATU (1.1 equiv per acid-PEG_12_-NHBoc) and 4-methylmorpholine (2.5 equiv per acid-PEG_12_-NHBoc) were added to the ELP solution slowly over the course of 10 min and left to react overnight. The reaction was then precipitated in ice-cold diethyl ether, pelleted, and vacuum-dried. The resulting protein (ELP-PEG) was dissolved in deionized (DI) water at 1 wt %, dialyzed against Milli-Q water for 3 days, and lyophilized to yield a white fibrous solid. Exo-norbornene was next conjugated onto the ELP-PEG via a HATU-mediated amidation reaction. Briefly, the lyophilized ELP-PEG was dissolved in anhydrous DMSO at 7.5 wt % and allowed to stir for 10 min before adding an equal volume of anhydrous DMF and stirring for an additional 10 min. In a separate flask, exo-norbornene acid (Sigma-Aldrich) was fully dissolved in an equal volume of DMF before adding HATU (1.1 equiv per exo-norbornene acid) and 4-methylmorpholine (2.5 equiv per exo-norbornene acid). Once dissolved, the reactants were stirred for 10 min before being added slowly to the ELP-PEG over the course of 10 min. The reaction was allowed to proceed overnight. The following day, the product was precipitated using ice-cold diethyl ether, pelleted, and vacuum-dried. The resulting ELP-norbornene was dissolved in water at 1 wt %, dialyzed against Milli-Q water for 3 days (molecular weight cutoff, 10 kDa), sterile-filtered through a 0.22-μm filter, and lyophilized to yield a white solid, which was stored at −20°C.

### Synthesis of aldehyde- and benzaldehyde-functionalized HA

Linear HA (100 kDa, sodium salt, LifeCore Biomedical) was functionalized in a two-step process, as described previously (*34*, *37*). Briefly, the carboxylic acid groups on HA were first modified with propargylamine using carbodiimide chemistry to form an intermediate HA-alkyne polymer. Second, the alkyne groups were modified with either 4-azidobenzaldehyde (Santa Cruz Biotechnology) to form HA-BZA or Ald-CH2-PEG3-azide (BroadPharm) to form HA-ALD.

HA-alkyne was prepared by first dissolving HA in MES buffer, consisting of 0.2 M MES hydrate (Sigma-Aldrich) and 0.15 M NaCl in Milli-Q water (pH 4.5), to a concentration of 1 wt % in a round bottom flask. Once dissolved, propargylamine (0.8 equiv to HA carboxylic acid groups; Sigma-Aldrich) was added to the HA, and the pH was immediately adjusted to 6 using NaOH. NHS (0.8 equiv to HA carboxylic acid groups; Thermo Fisher Scientific) and 1-ethyl-3-(3-dimethylaminopropyl)carbodiimide hydrochloride (EDC; 0.8 equiv to HA carboxylic acid groups; Thermo Fisher Scientific) were then added sequentially to the reaction vessel. The reaction was allowed to stir at RT overnight. The final solution was dialyzed against Milli-Q water for 3 days at 4°C with regular water changes (molecular weight cutoff, 10 kDa), sterile-filtered, and lyophilized to produce a white solid, which was stored at −20°C.

HA-ALD and HA-BZA were prepared from HA-alkyne in batches of 200 to 1000 mg. First, lyophilized HA-alkyne was dissolved to a final concentration of 1 wt % in 10× PBS [81 mM sodium phosphate dibasic, 19 mM sodium phosphate monobasic, and 60 mM sodium chloride in Milli-Q water (pH 7.4)] supplemented with β-cyclodextrin (1 mg/ml; Sigma-Aldrich). The solution was degassed with nitrogen (N_2_) for 30 min. Separate solutions of sodium ascorbate (4.52 mM, 0.18 equiv to HA carboxylic acid groups; Sigma-Aldrich) and copper(II) sulfate pentahydrate (0.24 mM, 0.0096 equiv to HA carboxylic acid groups; Sigma-Aldrich) were prepared by dissolving in Milli-Q water and degassing with N_2_. Next, these solutions were added sequentially to the vessel containing HA-alkyne via a syringe. Last, either azidobenzaldehyde (2.0 equiv alkyne groups) to produce HA-BZA or Ald-CH2-PEG3-azide (2.0 equiv alkyne groups) to produce HA-ALD was dissolved in a minimal amount of anhydrous DMSO (~300 mg/ml) and then added to the reaction vessel to produce HA-BZA and HA-ALD, respectively. The reaction was allowed to proceed for 24 hours while stirring at RT, and then an equal volume of 50 mM EDTA in Milli-Q water (pH 7.0; Thermo Fisher Scientific) was added to the reaction to chelate any remaining copper for 1 hour. Last, the solution was dialyzed against Milli-Q water for 3 days at 4°C with regular water changes (molecular weight cutoff, 10 kDa), sterile-filtered, and lyophilized to produce a white solid, which was stored at −20°C.

### Synthesis of tetrazine-functionalized HA

HA-tetrazine was synthesized in a similar process as previously described (*66*). Briefly, HA (100 kDa, sodium salt, LifeCore Biomedical) was dissolved in 0.1 M MES buffer (pH 7.0) at 1 wt %. Once dissolved, 1-hydroxybenzotriazole hydrate (2 equiv to the HA dimer unit; Thermo Fisher Scientific) was added to the HA and allowed to dissolve for 15 min. In a separate vial, tetrazine amine (TET-NH_2_; 2 equiv; Conju-Probe) was dissolved in a 6-ml solution of acetonitrile (MeCN) and DI water (5:1 v/v) to which EDC (2 equiv; Thermo Fisher Scientific) was added and allowed to dissolve. The solution was then added at a rate of 1 ml every 5 min into the dissolved HA solution and reacted overnight. After this time, the reaction was dialyzed for 2 days against a 10% MeCN solution, followed by 3 days against Milli-Q water (molecular weight cutoff, 10 kDa). The reaction products were then sterile-filtered using a 0.22-μm filter and lyophilized to produce a white solid, which was stored at −20°C.

### Ink mechanical characterization

Mechanical testing for all ink formulations was performed using an ARG2 stress-controlled rheometer (TA Instruments) and a 20-mm cone and plate geometry. All solutions were kept on ice before being loaded onto the rheometer stage. Inks were mixed to the final polymer concentration with and without competitor and catalyst and immediately added to the rheometer stage using a pipette (45 μl of solution). Gelation time sweeps were performed at a frequency of 1 rad/s and a strain of 1%. Frequency sweeps were performed between 0.1 and 100 rad/s at a strain of 1%. The final shear modulus was taken from the linear region of the frequency sweep at an angular frequency of 1 rad/s. Shear-thinning tests were performed at shear rates ranging from 0.1 to 100 s^−1^, and a steady-state viscosity was confirmed for each data point (fig. S15). Stress relaxation tests were performed at a strain rate of 10% for 10 hours. All measurements were collected at 23°C.

Yield stress measurements were conducted on 45 μl of samples using a 20-mm cone and plate geometry as above. Samples were first cross-linked at 23°C during a 30 min time sweep at a frequency of 1 rad/s and a strain of 1%. Next, a multiple creep test was performed to determine the yield stress (*67*, *68*). To do so, the % strain (γ) was measured for an applied fixed stress (σ) over the course of 3 min, and then, the material was allowed to relax (σ = 0) for 15 min. This cycle was repeated such that each subsequent stress was twice as high as the previous stress (e.g., σ = 1, 2, 4, and 8 Pa), up until a stress that induced failure of the material. From these data, the yield stress was taken to be the stress that induces irrecoverable deformation (when the % strain no longer returns to 0 during the relaxation stage, here taken as γ > 0.5%).

Modulus recovery experiments (Fig. 2B) were performed on HELP hydrogels (1 wt % ELP-HYD, 0.5 wt % HA-ALD, and 0.5 wt % HA-BZA) cast into 8-mm-diameter–by–0.8-mm-depth silicone molds within six-well plates. After the appropriate gel components were mixed together, the solution was pipetted into each mold individually, and hydrogel cross-linking was allowed to occur over 30 min at 37°C. For *t* = 0 hour measurements, gels were then removed from their molds and placed onto the rheometer stage. For *t* = 24-hour samples, 5 ml of prewarmed PBS was added to each well after the 30 min cross-linking period, and samples were incubated at 37°C for 24 hours. Rheological characterization was performed using an 8-mm parallel plate geometry. Both the geometry and rheometer stage were affixed with a thin section of sandpaper to prevent hydrogel slippage during measurements. Samples were placed on the rheometer stage on top of the fixed sandpaper, and the geometry head was lowered onto the gel. Once the normal force reached a value of 0.1 N, a frequency sweep was immediately performed. The final shear modulus was derived from the linear region of the frequency sweep at an angular frequency of 1 rad/s. Data were normalized to the average value of the shear storage modulus for gels at *t* = 0 hours without any competitor added.

### Ink and support bath preparation

Lyophilized ELP-HYD, HA-ALD, and/or HA-BZA were dissolved overnight at 4°C in isotonic 10× PBS [81 mM sodium phosphate dibasic, 19 mM sodium phosphate monobasic, and 60 mM sodium chloride in Milli-Q water (pH 7.4)] to working concentrations of 4, 2, and 2 wt %, respectively. The competitor (hydrazinoacetic acid; BOC Sciences) and catalyst 1 [2-(aminomethyl)benzimidazole; Sigma-Aldrich] were purchased and used as received. Catalyst 2 was synthesized as previously described (*39*). The catalysts and competitor were dissolved in 10× PBS to working concentrations of 100 and 200 mM, respectively. All solutions were kept on ice. To form an ink, the competitor and catalyst were added to ELP-HYD to the appropriate concentration (typically, 20 and 10 mM, respectively, in the final ink solution). Either food coloring or fluorescent microspheres was then added to aid in visualization of printed structures. Last, the HA component was added to the mixture, and then the ink solution was quickly added to the print syringe (2.5-ml gastight Hamilton syringe prefitted with a 27-gauge blunt needle).

LifeSupport (FluidForm Inc.) is the commercialized gelatin microparticle support bath produced using the FRESH 2.0 process (*5*), and baths were prepared using the manufacturer’s recommendations. Briefly, lyophilized LifeSupport was hydrated using sterile, cold PBS for 10 min. Then, the hydrated particles were centrifuged (2000*g* for 5 min), and the supernatant was removed via aspiration. The compacted slurry was added to custom-made polycarbonate containers, centrifuged to remove any bubbles, and kept on ice before use. Printing was performed using 27-gauge needles with a print speed of 23 mm/s, extrusion width of 0.21 mm, and layer height of 0.084 mm for all structures (cellular and acellular) presented within the manuscript. 3D printed models were sliced using Simplify3D.

### Cell culture

H-ras–transformed human MCF10AT cells expressing an H2B-GFP (green fluorescent protein) fusion were graciously provided by J. Liphardt’s laboratory (Stanford University). The cells were maintained as previously described (*69*). Briefly, cells were expanded in Dulbecco’s modified Eagle’s medium/F12 (Thermo Fisher Scientific) containing 5% horse serum (Gibco), recombinant human epidermal growth factor (20 ng/ml; Thermo Fisher Scientific), recombinant human insulin (10 μg/ml; Sigma-Aldrich), hydrocortisone (0.5 mg/ml; Sigma-Aldrich), cholera toxin (100 ng/ml; Sigma-Aldrich), and 1% penicillin-streptomycin (Thermo Fisher Scientific). Cells were passaged every other day or upon reaching 70 to 90% confluency through dissociation with TrypLE Express (Thermo Fisher Scientific). For experiments with TGF-β, recombinant human TGF-β1 (10 ng/ml; Thermo Fisher Scientific) was added to the cell culture medium starting on day 0. Primary breast CAFs were purchased and cultured according to the manufacturer’s recommendations (Cell Biologics). For coculture of dual–cell-type prints, MCF10AT and CAF medium were mixed in a 1:1 ratio.

### 3D bioprinting

All bioink materials for cell studies were dissolved in sterile 10× PBS to their final working concentrations. Before printing, cells were trypsinized, counted, pelleted, and resuspended in 4 wt % ice-cold ELP-HYD at a cell density of 4 × 10^6^ ml^−1^. Next, the appropriate amounts of sterile-filtered catalyst and competitor were added to the resuspended cells (table S1). Last, the HA component (2 wt % 50:50 HA-ALD:HA-BZA) was mixed into the cell resuspension on ice. The ink mixture was then immediately added to the barrel of a 2.5-ml Hamilton syringe fitted with a 27-gauge needle. Cell-containing bioinks were typically printed as 8 mm–by–1 mm disks (unless otherwise noted; table S1) into sterile LifeSupport baths hydrated with PBS. Printed structures were incubated for at least 1.5 hours at RT to allow for catalyst and competitor diffusion out of the construct (table S1). Then, the support bath was melted by placing the entire container in an incubator (37°C, 5% CO_2_) for 20 min. After the support bath was completely liquified, the structure was removed from the bath and washed thoroughly with sterile, prewarmed PBS. After washing with PBS, the appropriate cell medium was added on top of the printed structure and the cell-laden constructs were incubated at 37°C and 5% CO_2_. The medium was changed once daily for the culture period.

### Dual-material bioprinter modification and printing

Printing was carried out on a custom-built dual-extruder bioprinter modified from a MakerGear M2 Rev E plastic 3D printer, as previously described (*44*). Briefly, the entire thermoplastic extruder was removed and replaced with a mount designed to hold two Replistruder 4 syringe pumps, and the control board was replaced with a Duet 2 WiFi board with RepRapFirmware (*70*). For dual material bioprinting, two Hamilton syringes fitted with 27-gauge needles were filled with their respective bioinks. To determine the boundaries of multimaterial prints, fluorescent microspheres with different emission/absorption wavelengths were included in each bioink at a concentration of 10 ng/ml. Printing needles were aligned manually by measuring the physical distance between the two needle tips and recording the tool offsets using G-Code commands (G10) built into the printer’s RepRapFirmware. When the tools are switched, a G-Code script is automatically executed that removes the active tool from the support bath, switches the active tool, moves the new tool to its origin (the measured offset of the other tool), and then resumes printing with the new tool.

### Hydrogel erosion

HELP inks formulated with 0.75% ELP-HYD/0.25% ELP-HYD-Cy5/1% HA (either ALD-only, 50:50 ALD:BZA, or BZA-only)/20 mM competitor/10 mM catalyst were printed into 6-mm disks within a LifeSupport bath. Printed disks were incubated for 1.5 hours at RT to allow for catalyst and competitor diffusion. Then, the support bath was melted, and the printed disks were removed from the bath and rinsed with sterile, prewarmed PBS. Printed disks were then covered in 1 ml of PBS and incubated at 37°C and 5% CO_2_. At the indicated timepoints (fig. S7), all the PBS was removed for fluorescent measurement and replaced with fresh PBS. The fluorescence intensity of the ELP-HYD-Cy5 in the removed PBS was measured for each sample on a plate reader using an excitation of 650 nm and an emission reading at 670 nm. After 14 days, the printed hydrogels were completely degraded using PBS supplemented with elastase (200 U/ml; GoldBio), hyaluronidase (2000 U/ml; Sigma-Aldrich), and 3 mM EDTA. The fluorescence of the degraded hydrogel was measured and used to calculate the total amount of fluorescence intensity remaining in each hydrogel to normalize the data.

### Quantitative polymerase chain reaction

For quantitative polymerase chain reaction (qPCR) analysis, MCF10AT cells were cultured in 40 μl HELP hydrogels within 8-mm-diameter–by–0.8-mm-thick circular silicone molds. To collect cell lysates, the HELP matrix was first dissociated using a solution containing PBS supplemented with elastase (200 U/ml; GoldBio), hyaluronidase (2000 U/ml; Sigma-Aldrich), and 3 mM EDTA for 30 min at 37°C. Next, the dissociated gels were resuspended in 500 μl of TRIzol (Invitrogen) and disrupted via probe sonication [Heilscher UP50H, 50% amplitude (25 W), 30-kHz frequency, 0.5 cycle]. mRNA was then purified from lysates using a phenol-chloroform extraction using a phase lock gel (Quantabio 5PRIME), followed by an isopropyl alcohol precipitation, and then two washes of 70% ethanol with centrifugation steps between each wash (18,500*g* at 4°C for 10 min). After decanting the final ethanol wash, samples were dried and resuspended in nuclease-free water. mRNA (200 ng) was reverse-transcribed using aHigh-Capacity cDNA Reverse Transcription Kit (Applied Biosystems) and then diluted 10-fold with nuclease-free water. qPCR was performed on 6.6 μl of diluted cDNA per gene target mixed with 0.9 μl of 5 μM forward and reverse primer pair solution and 7.5 μl of the Fast SYBR Green Master Mix (Applied Biosystems). The reaction mixture was run on aStepOnePlus Real Time PCR System (Applied Biosystems), and the mRNA expression was analyzed using the ΔC_T_ method.

### Microscopy

Fluorescence microscopy was performed using either a Leica SPE confocal microscope with a 10× air objective or 63× oil immersion objective or a Leica THUNDER imager with a 2.5× objective or 10× objective. Cell-laden hydrogels used for fluorescence microscopy were cast into 4-mm-diameter–by–0.8-mm-depth silicone molds plasma-bonded to glass coverslips. Bioprinted structures were also imaged using fluorescence microscopy by either sandwiching printed constructs between glass coverslips or placing 8-mm-diameter printed disks within 8-mm-diameter–by–0.8-mm-depth silicone molds bonded to glass coverslips. Individual samples were prepared for fluorescence microscopy by fixation with 4% paraformaldehyde in PBS for 30 min at RT. The samples were then washed three times with PBS for 15 min. Cell membranes were permeabilized with 0.1% Triton X-100 in PBS (PBST) for 1 hour. Nuclei and F-actin were stained by incubation with 4′,6-diamidino-2-phenylindole (1 μg/ml; Thermo Fisher Scientific) and phalloidin–tetramethyl rhodamine B isothiocyanate (TRITC-phalloidin; 0.2 μg/ml; Sigma-Aldrich) in PBST for 1 hour at RT. Staining was followed by three 20-min washes in PBST. Last, samples were mounted onto coverslips with a ProLong Gold Antifade reagent (Thermo Fisher Scientific) and allowed to cure for 48 hours before imaging.

Cell viability was assessed by staining with calcein AM to label live cells and ethidium homodimer to label dead cells using a Live/Dead staining kit (Thermo Fisher Scientific). For competitor cytotoxicity assays, MCF10AT cells were encapsulated in HELP hydrogels (1% ELP-HYD/0.5% HA-ALD/0.5% HA-BZA) containing varying amounts of competitor (0 to 50 mM), and then cell viability was measured after 24 hours in culture. After 24 hours, cells were stained with calcein AM (2 μM) and ethidium homodimer (4 μM) in PBS for 20 min at 37°C. Cells were then imaged directly using a 10× air objective. For bioprinted constructs, MCF10AT cells were printed in HELP bioinks into 8-mm-diameter disks. After removal from the support bath, printed disks were washed with prewarmed, sterile PBS and then placed in 12-well plates. Next, cell viability was evaluated by incubating the printed disks with calcein AM (2 μM) and ethidium homodimer (4 μM) in PBS for 20 min at 37°C. Printed gels were then imaged directly in the wells in PBS using a 10× air objective.

### Image analysis

MCF10AT cell cluster size and circularity analysis were performed using ImageJ. All cells were stained for F-actin with TRITC-phalloidin, and nuclei were visualized using the endogenously expressed H2B-GFP protein. *Z* stacks were taken using a 10× objective with 10-μm spacing between slices for both printed and cast hydrogels. At least three images were taken in different areas of each hydrogel. Shape analysis was performed by thresholding the image, removing objects with areas less than 500 μm^2^, and calculating the 2D projected area and perimeter of each cluster. Circularity was calculated as 4π×area/perimeter^2^, where 1 indicates a perfect circle. For printed lattices, window printability was determined by thresholding the image and then measuring the area and perimeter of each window within the lattice. Printability (Pr) was then calculated as perimeter^2^/16×area, where 1 indicates a perfect square (*71*).

### Statistical analysis

Statistical analyses for this study were performed using GraphPad Prism version 9 software. Details of specific statistical methods for each figure are included within the figure captions. For all studies, not significant (ns; *P* > 0.05), *P < 0.05, **P < 0.01, ***P < 0.001, and ****P < 0.0001.
