# Supplementary material for: 3D bioprinting of dynamic hydrogel bioinks enabled by small molecule modulators
Source: Sci Adv. 2023 Mar 31;9(13):eade7880. doi: 10.1126/sciadv.ade7880 (PMC10065439; doi:10.1126/sciadv.ade7880)
Supplement: Supplementary file 1 — Figs. S1 to S15 Tables S1 and S2 [file sciadv.ade7880_sm.pdf]

Science Advances
AAAS

Supplementary Materials for

3D bioprinting of dynamic hydrogel bioinks enabled by small molecule modulators

Sarah M. Hull *et al.*

Corresponding author: Sarah C. Heilshorn, heilshorn@stanford.edu

Sci. Adv. **9**, eade7880 (2023)
DOI: 10.1126/sciadv.ade7880

This PDF file includes:

Figs. S1 to S15
Tables S1 and S2

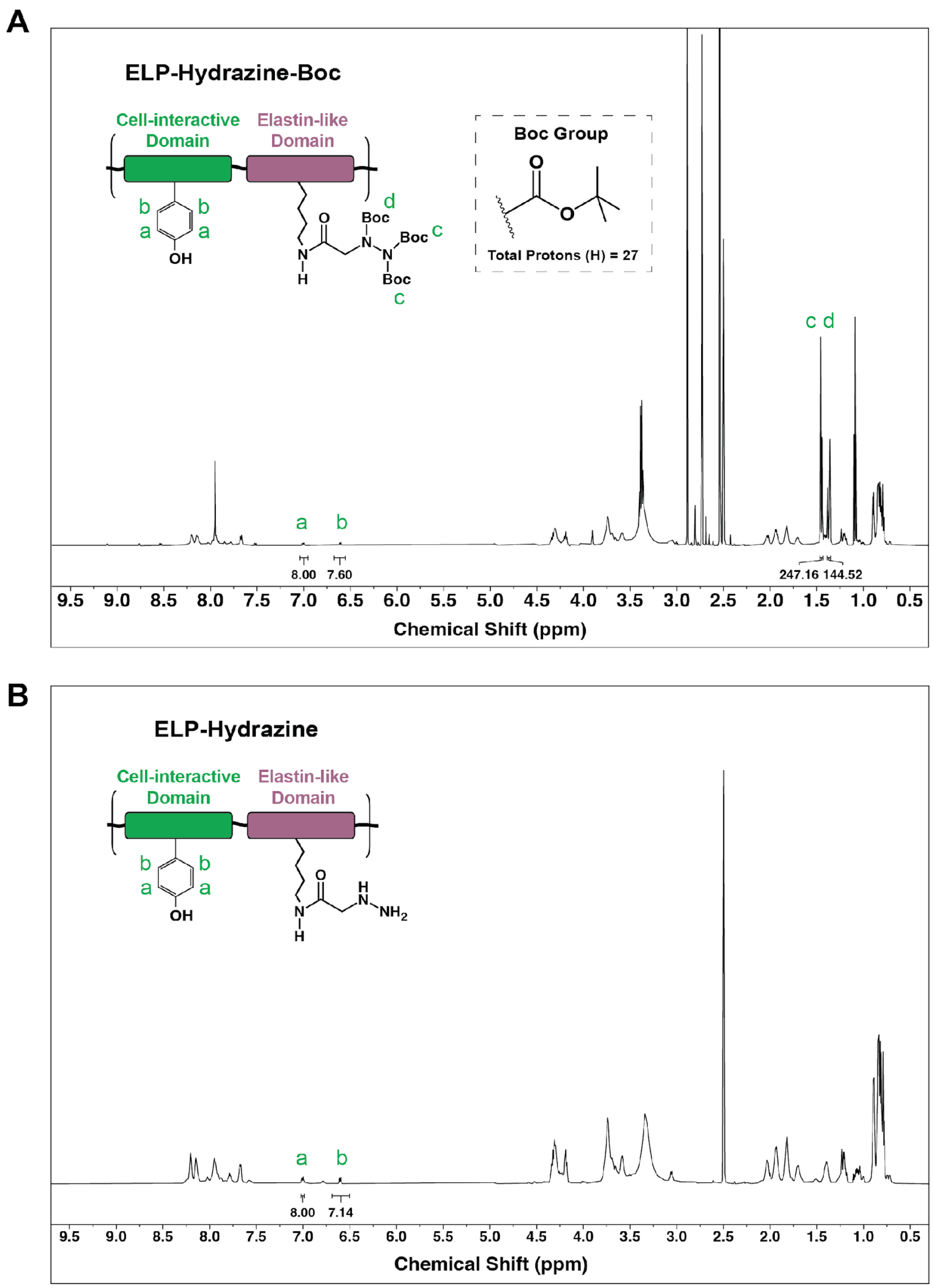

Fig. S1. Elastin-like protein (ELP) modification with hydrazine functional groups.

(**A**) Representative proton nuclear magnetic resonance (^{1}H NMR, DMSO solvent) of the intermediate ELP-Hydrazine-Boc, showing ~100% modification of ELP. The degree of modification is calculated by normalizing to the tyrosine peaks from ELP. (**B**) The butyloxycarbonyl (Boc) protecting group is completely removed from the hydrazine in the final

ELP-Hydrazine polymer through an acid-mediated deprotection step. Representative ^{1}H NMR (DMSO solvent) of ELP-Hydrazine showing full Boc removal (δ = 1.46 and 1.39 ppm).

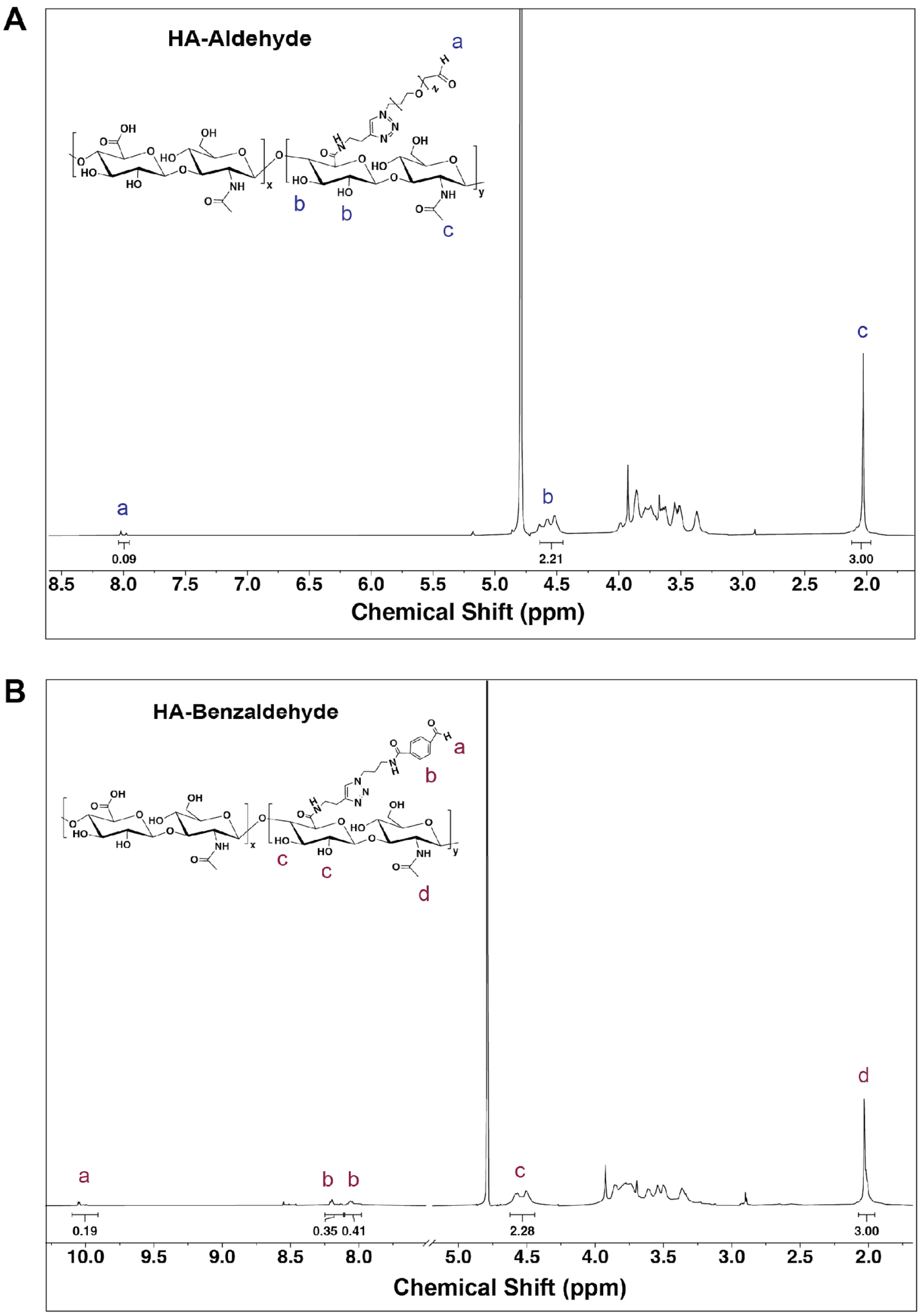

Fig. S2. Hyaluronic acid (HA) modification with aldehyde or benzaldehyde functional groups.

(**A**) Representative ^1H NMR (D_2O solvent) of ~9% modified HA-Aldehyde. (**B**) Representative ^1H NMR (D_2O solvent) of ~19% modified HA-Benzaldehyde.

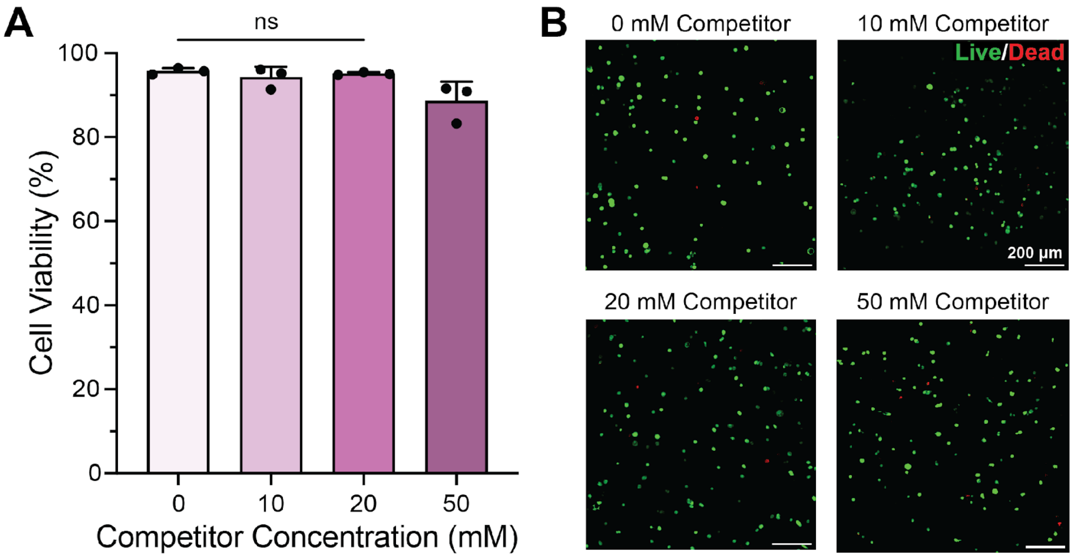

Fig. S3. Cytotoxicity of small molecule competitor.

(**A**) MCF10AT cells remain viable after 24 h of competitor exposure. Cells were encapsulated in HELP hydrogels (1% ELP-HYD/0.5% HA-ALD/0.5% HA-BZA) containing varying amounts of competitor, and then cell viability was measured after 24 h in culture. Addition of the competitor up to 20 mM did not significantly reduce cell viability (N=3, mean ± SD, ordinary one-way ANOVA with Tukey multiple comparisons correction, ns = not significant). (**B**) Representative Live/Dead image of MCF10AT cells in 3D HELP hydrogels with varying amounts of competitor after 24 h.

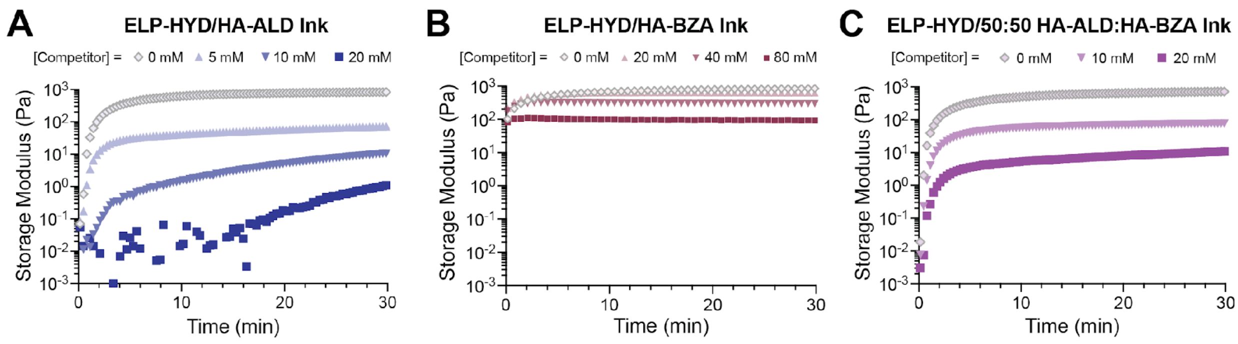

Fig. S4. Rheological characterization of HELP hydrogels containing competitor.
HELP hydrogels formulated with (**A**) 1% HA-Aldehyde/1% ELP-Hydrazine, (**B**) 1% HA-Benzaldehyde/1% ELP-Hydrazine, or (**C**) 0.5% HA-Aldehyde/0.5% HA-Benzaldehyde/1% ELP-Hydrazine with varying amounts of competitor form hydrogels suitable for printing within 30 min.

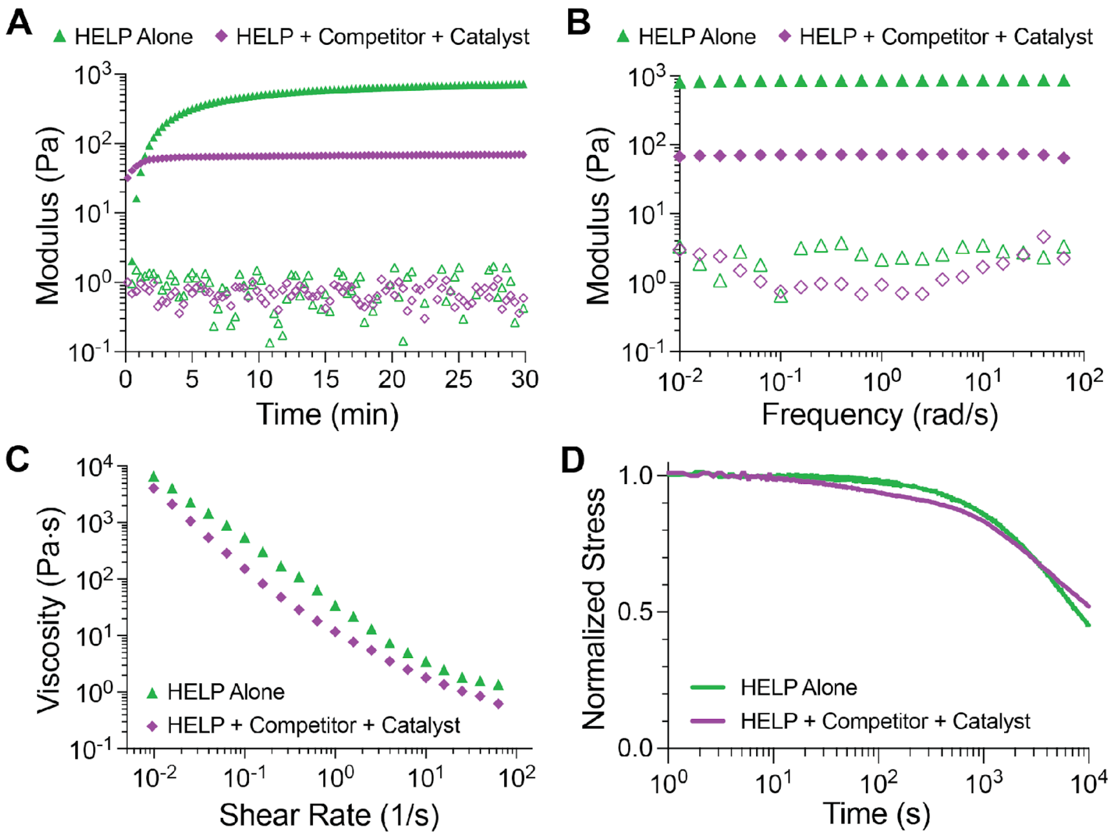

Fig. S5. Rheological properties of HELP inks containing competitor and catalyst.
(**A**) Time sweep, (**B**) frequency sweep, (**C**) shear rate sweep, and (**D**) stress relaxation of HELP hydrogel inks (1 wt% ELP-HYD, 0.5 wt% HA-ALD, and 0.5 wt% HA-BZA) with and without competitor (20 mM) and catalyst (10 mM). The addition of the competitor and catalyst decreases the gelation time (**A**), decreases the storage modulus (**B**), and increases the hydrogel's ability to shear thin (**C**), but does not affect the hydrogel's stress relaxation behavior (**D**).

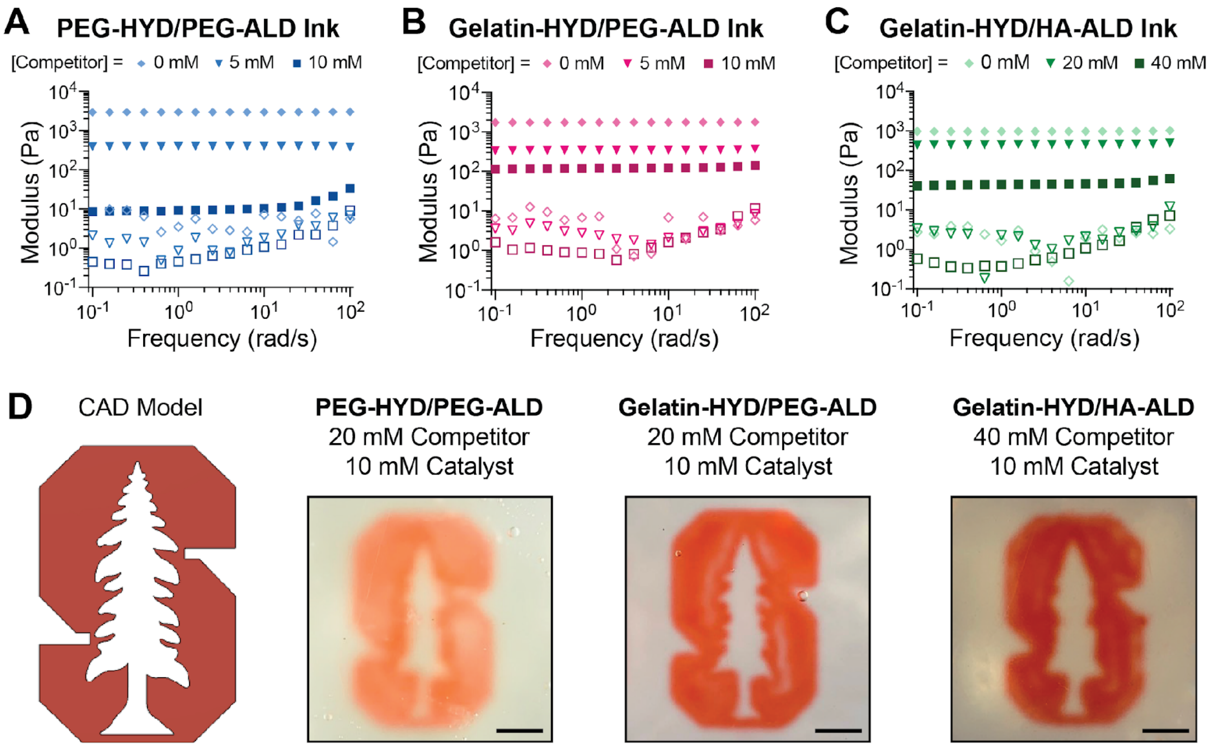

Fig. S6. Expanding the library of hydrazone-crosslinked, dynamic inks.

(**A**) An 8-arm, 40 kDa PEG was functionalized with either aldehyde or hydrazine groups. When 3 wt% PEG-HYD and 3 wt% PEG-ALD are mixed, they form hydrogels with storage moduli of ~3000 Pa. Addition of the competitor decreases the storage modulus in a dose-dependent manner. (**B**) Gelatin can also be functionalized with hydrazine groups. When 4 wt% Gelatin-HYD and 3 wt% PEG-ALD are mixed, they form hydrogels with storage moduli of ~2000 Pa. Addition of the competitor decreases the storage modulus in a dose-dependent manner. (**C**) 3 wt% Gelatin-HYD and 1 wt% HA-ALD are mixed to form hydrogels with storage moduli of ~1000 Pa. Addition of the competitor decreases the storage modulus in a dose-dependent manner. Storage moduli are filled symbols, while loss moduli are open symbols. (**D**) Each of these hydrazone inks (PEG-HYD/PEG-ALD, Gelatin-HYD/PEG-ALD, and Gelatin-HYD/HA-ALD) was printable with the addition of competitor and catalyst into complex shapes, including the Stanford logo. Scale bars = 4 mm.

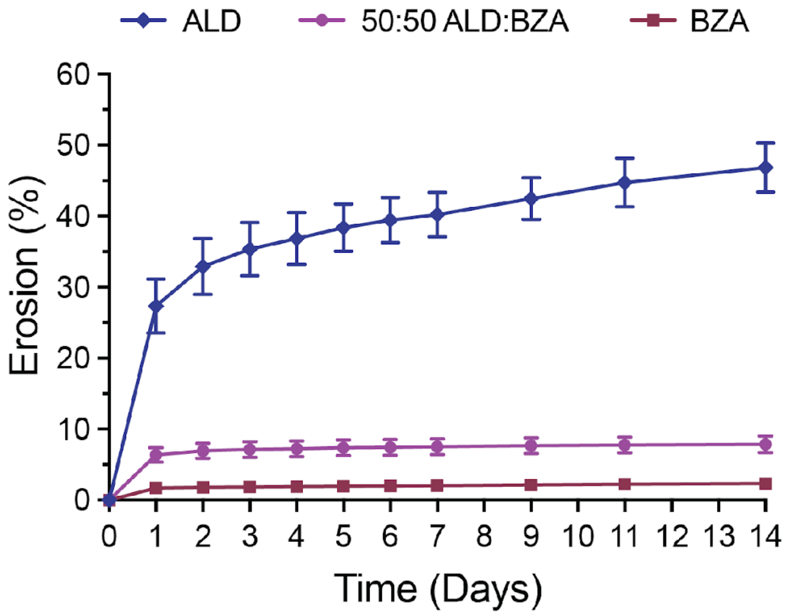

Fig. S7. Erosion kinetics of printed HELP ink materials.

Erosion of printed HELP inks with varying ratios of ALD:BZA (i.e. ALD-only, 50:50 ALD:BZA, and BZA-only) at 37 °C, as determined by measuring the fluorescence intensity of the release of Cy5-conjugated ELP-HYD. All HELP formulations included 1 wt% ELP, 1 wt% HA, 20 mM of competitor, and 10 mM of catalyst.

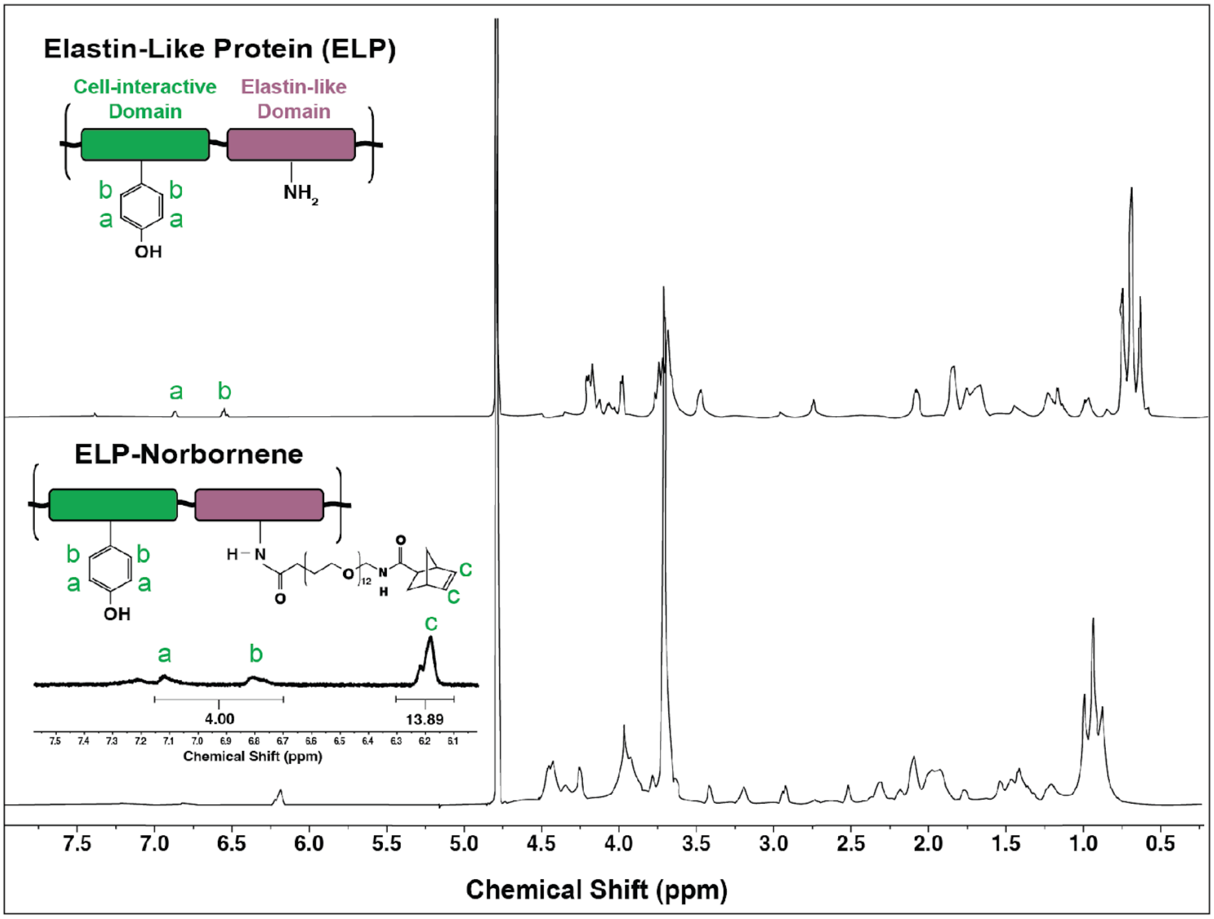

Fig. S8. ELP modification with norbornene functional groups.

Representative ^1H NMR (D_2O solvent) of ELP (top) and ELP modified with norbornene (bottom). Integration of the proton signal of the alkene hydrogens on the norbornene ring (δ= 6.2, 2H) relative to the protons of the four available tyrosine peaks (δ= 7.25 and 6.75, 4H) showed a 99.2% modification.

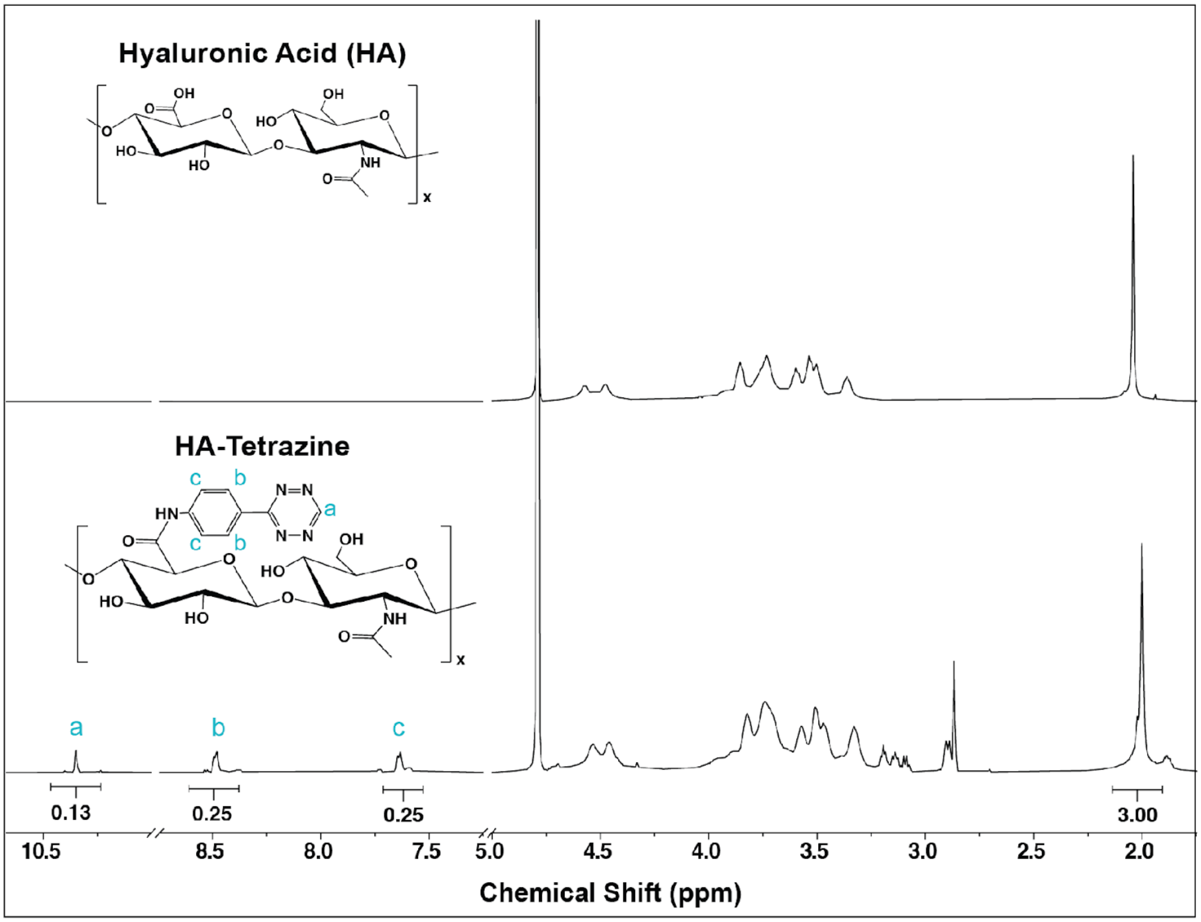

Fig. S9. HA modification with tetrazine functional groups.

Representative ^1H NMR in D_2O of HA (top) and ~13% modified HA-tetrazine (bottom). The degree of modification was determined through integration of the proton signal of the tyrosine peaks (δ= 8.5 and 7.6, 4H) and tetrazine peak (δ= 10.3, 1H) relative to that of the methyl groups on the N-acetylglucosamine of the HA backbone (δ= 1.8, 3H).

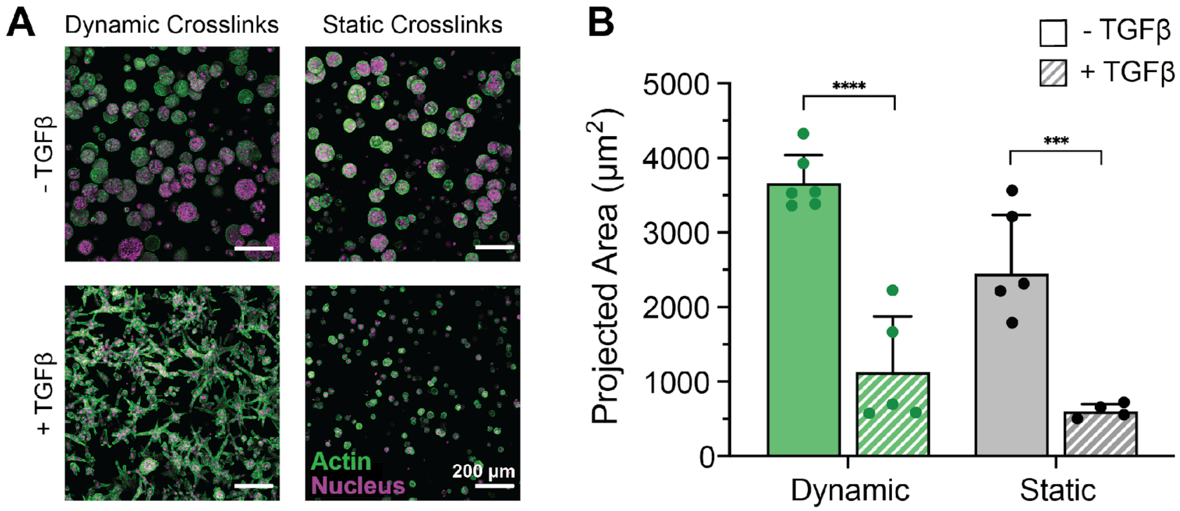

Fig. S10. Addition of TGFβ drives morphological changes in breast cancer cells cultured within HELP matrices.

(**A**) Representative 10x images of MCF10AT cells grown in HELP-Dynamic and HELP-Static for 6 d, with and without the addition of 10 ng/mL TGFβ to the medium. (**B**) Quantification of 2D projected area of cell clusters in HELP-Dynamic and HELP-Static, where the addition of TGFβ decreases cluster size, consistent with previous reports of TGFβ decreasing proliferation (*48*). (N=4-6, mean ± SD, ordinary one-way ANOVA with Tukey multiple comparisons correction, ***$p<0.01$, ****$p<0.0001$).

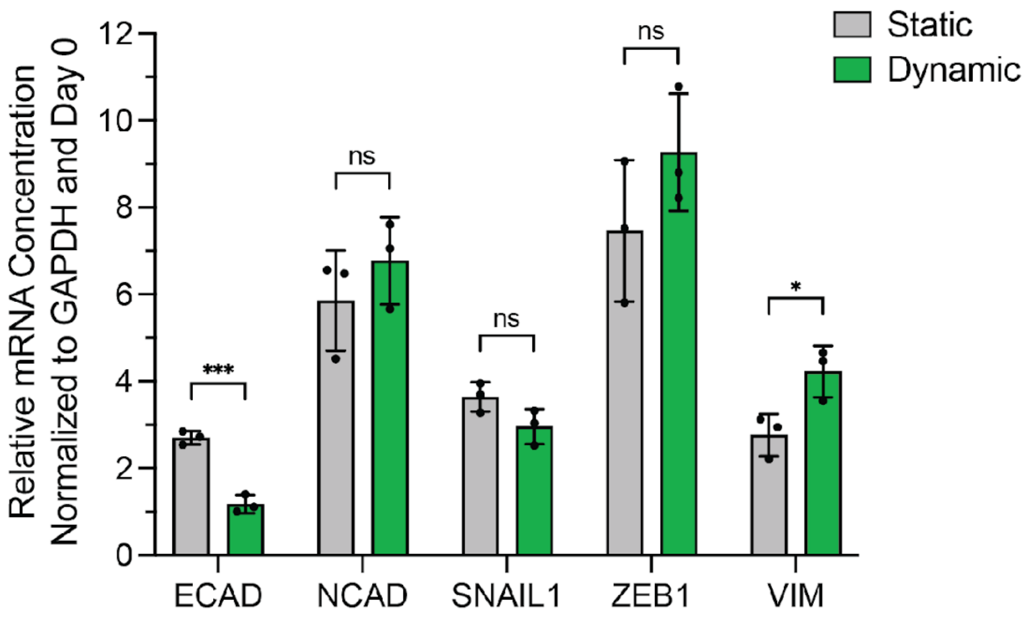

Fig. S11. Gene expression of EMT markers for MCF10AT cells cultured in HELP matrices. qPCR quantification of mRNA-level EMT marker expression in MCF10AT cells cultured within HELP-Static or HELP-Dynamic for 6 d with the addition of 10 ng/mL TGFβ. Data are normalized to GAPDH gene expression and respective marker expression on Day 0 prior to cell encapsulation. (N=3, mean ± SD, unpaired two-tailed Student's t-test comparing HELP-Static and HELP-Dynamic for the indicated marker, *$p<0.05$, ***$p<0.001$, ns = not significant).

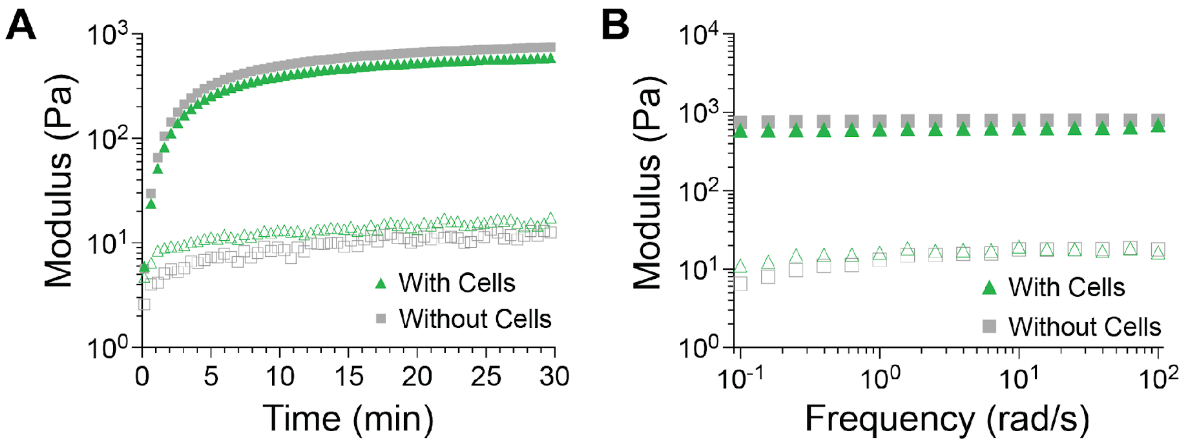

Fig. S12. Presence of cells does not affect gelation kinetics or final modulus of HELP hydrogels.

(**A**) Time sweep and (**B**) frequency sweep of HELP inks (1% ELP-HYD/0.5% HA-ALD/0.5% HA-BZA) with and without 0.5x10^6 MCF10AT cells/mL. Storage moduli are filled symbols, while loss moduli are open symbols.

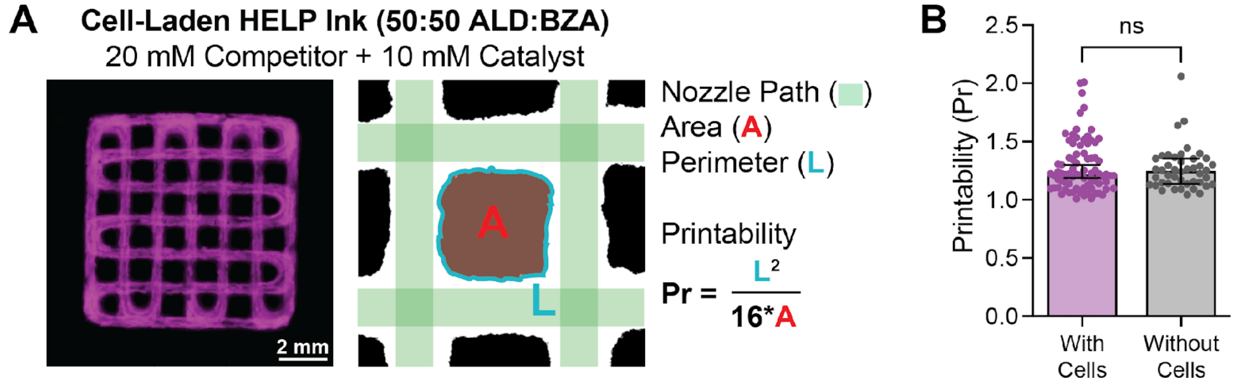

Fig. S13. Printability of acellular HELP inks and cell-laden HELP bioinks.

(**A**) Printed lattices containing $2x10^6$ MCF10AT cells/mL within the optimized HELP ink (1% ELP-HYD/0.5% HA-ALD/0.5% HA-BZA, 10 mM catalyst, 20 mM competitor). A small amount of ELP-HYD-Cy5 was blended with ELP-HYD to aid in visualization of the printed bioink. The shape fidelity was determined by quantifying the window printability (*Pr*), which compares the perimeter (*L*) to the area (*A*) of each individual printed window within the lattice to determine its similarity to a perfect square, as described previously (*71*). (**B**) The quantified printability for lattices printed with and without cells was not significantly different and was close to 1 (a perfect square). (N=3, n=44-78 printed windows, mean ± 95% confidence interval, two-tailed Mann-Whitney test, ns = not significant).

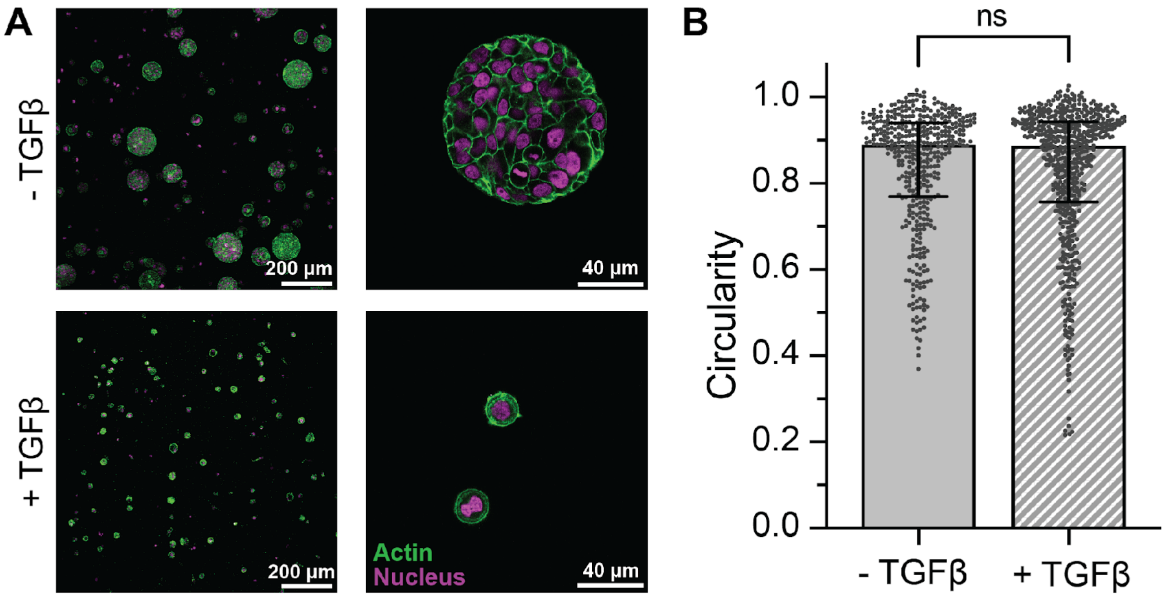

Fig. S14. MCF10AT cells cultured within HELP-Dynamic containing RDG remain circular upon addition of TGFβ.

(**A**) Representative images of MCF10AT cells encapsulated within HELP matrices containing the non-integrin-binding RDG peptide sequence at 10x (left) and 63x (right) magnification after 6 d in culture. (**B)** Addition of 10 ng/mL TGFβ to the cell culture medium does not induce cell spreading in HELP-RDG, as quantified by cell circularity. Representative data from one biological replicate experiment with median and interquartile ranges is shown. (n=444-633 cell clusters, two-tailed Mann-Whitney test, ns = not significant, N=3 biological replicates).

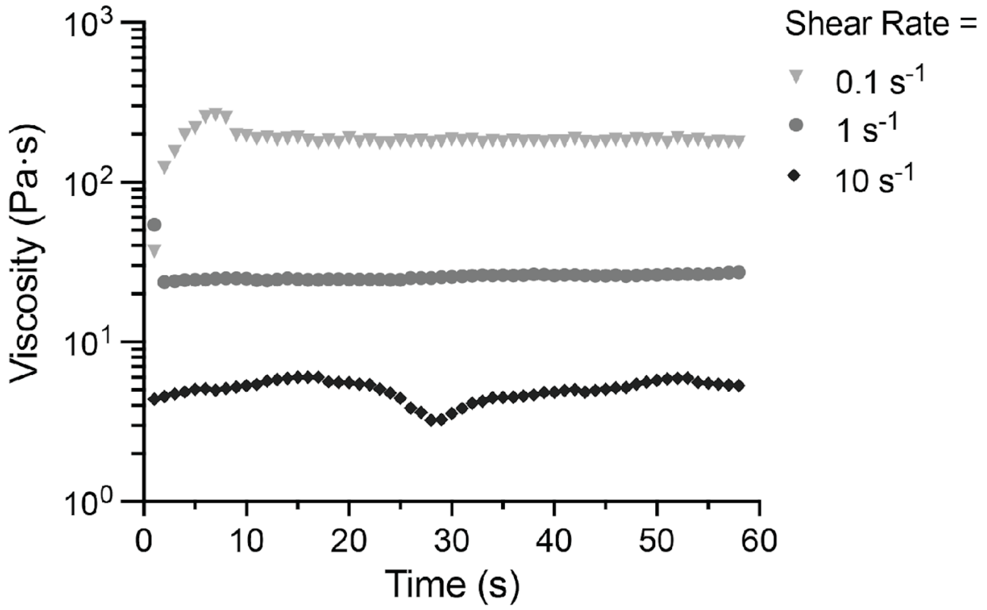

Fig. S15. Shear-thinning of HELP hydrogels containing catalyst.

Representative plot for a HELP formulation containing 1% ELP-HYD/1% HA-ALD/10 mM catalyst at various shear rates to demonstrate that a steady-state viscosity was reached for each data point presented in Fig. 2D.

**Figure**	**Print Description**	**Polymers in Ink**	**Competitor Concentration**	**Catalyst Concentration**	**Support Bath Condition**
2E (left)	Lattice	1 wt% ELP-HYD/ 1 wt% HA-ALD	0 mM	0 mM	None (Air)
2E (right)	Lattice	1 wt% ELP-HYD/ 1 wt% HA-ALD	20 mM	10 mM	None (Air)
3A (top)	Lattice	1 wt% ELP-HYD/ 1 wt% HA-ALD	20 mM	10 mM	1.5 h; RT
3A (middle); S13	Lattice	1 wt% ELP-HYD/ 0.5 wt% HA-ALD/ 0.5 wt% HA-BZA	20 mM	10 mM	1.5 h; RT
3A (bottom)	Lattice	1 wt% ELP-HYD/ 1 wt% HA-BZA	40 mM	10 mM	1.5 h; RT
5A/B	MCF10AT-laden 8-mm disk	1 wt% ELP-HYD/ 0.5 wt% HA-ALD/ 0.5 wt% HA-BZA	20 mM	10 mM	1.5 h; RT
5C	Dual material alternating lines of ELP-RDG and ELP-RGD	1 wt% ELP-HYD/ 0.5 wt% HA-ALD/ 0.5 wt% HA-BZA	20 mM	10 mM	1.5 h; RT
S4 (left)	Stanford logo	3 wt% PEG-HYD/ 3 wt% PEG-ALD	20 mM	10 mM	Overnight; 4 °C
S4 (middle)	Stanford logo	4 wt% Gelatin-HYD/ 3 wt% PEG-ALD	20 mM	10 mM	Overnight; 4 °C
S4 (right)	Stanford logo	3 wt% Gelatin-HYD/ 1 wt% HA-ALD	40 mM	10 mM	Overnight; 4 °C
S7	6-mm disk	1 wt% ELP-HYD/ 1 wt% HA (ALD, BZA, or 50:50 ALD:BZA)	20 mM	10 mM	1.5 h; RT

Table S1. Ink formulations and support bath conditions for all printed structures presented within the manuscript.

Primer	Sequence
hECAD-Fwd	CGAGAGCTACACGTTCACGG
hECAD-Rev	GGCCTTTTGACTGTAATCACACC
hSNAIL1-Fwd	TCGGAAGCCTAACTACAGCGA
hSNAIL1-Rev	AGATGAGCATTGGCAGCGAG
hZEB1-Fwd	GATGATGAATGCGAGTCAGATGC
hZEB1-Rev	ACAGCAGTGTCTTGTTGTTGT
hNCAD-Fwd	TCAGGCGTCTGTAGAGGCTT
hNCAD-Rev	ATGCACATCCTTCGATAAGACTG
hVimentin-Fwd	GACGCCATCAACACCGAGTT
hVimentin-Rev	CTTTGTCGTTGGTTAGCTGGT

Table S2. Primers used for qPCR.
